# Pharmacovigilance analysis of central nervous system adverse events associated with sevoflurane and drug interactions: a disproportionality study based on the FDA adverse event reporting system (FAERS) database

**DOI:** 10.3389/fphar.2026.1789072

**Published:** 2026-06-30

**Authors:** Hong Yu, Bing Wang

**Affiliations:** Department of Anaesthesia, Suizhou Hospital, Hubei University of Medicine, Suizhou, Hubei, China

**Keywords:** anesthesia safety, central nervous system adverse events, disproportionality analysis, drug-drug interactions, FAERS database, movement disorders, pharmacovigilance, sevoflurane

## Abstract

**Introduction:**

Sevoflurane is rarely administered as monotherapy in clinical anesthesia practice, yet the safety profile of its drug-drug interactions (DDIs) remains incompletely characterized in real-world settings. To systematically identify and quantify disproportionality signals for central nervous system (CNS) adverse events associated with sevoflurane combinations using the FDA Adverse Event Reporting System (FAERS) database.

**Methods:**

Reports from 2004 Q1 to 2025 Q2 listing sevoflurane as primary suspect were extracted and deduplicated. Disproportionality analysis employed Reporting Odds Ratio (ROR) and Proportional Reporting Ratio (PRR) to detect signals, supplemented by Bayesian methods (EBGM and IC) for multi-method validation. A hierarchical approach progressed from broad CNS adverse event screening to drug class-level and individual drug-level analyses. Interaction Reporting Odds Ratio (IOR) models quantified synergistic interaction signals under both PS-restricted and role-independent frameworks, with the Ω shrinkage measure applied as a Bayesian validation method for all IOR signals.

**Results:**

Among 4,129 sevoflurane reports (2,903 concomitant use, 1,226 quasi-monotherapy), multiple significant signals emerged. Intravenous anesthetics and opioid analgesics demonstrated the highest signal rates (44.4% each). Movement disorders, particularly dystonia, exhibited the strongest signals across multiple drug classes. Morphine combination with sevoflurane showed a notably high dystonia signal (ROR: 66.64). IOR analysis identified supra-additive interactions: morphine-dystonia (IOR: 9.46), fentanyl-dystonia (IOR: 2.96), and propofol-confusional state (IOR: 2.56). Ω shrinkage validation confirmed fentanyl-dystonia and propofol-confusional state as true synergistic signals. The role-independent analysis identified seven additional supra-additive IOR signals, with morphine-dystonia retaining significance across both frameworks. Sensitivity analyses confirmed the robustness of the core signals.

**Conclusion:**

Sevoflurane DDIs might be associated with multiple CNS adverse event signals, particularly movement and consciousness disorders. Dual-method validation using IOR and Ω shrinkage measure enhanced signal discrimination and reduced false-positive risk. These findings warrant clinical vigilance in vulnerable populations and prospective validation studies.

## Introduction

Sevoflurane, a halogenated volatile anesthetic agent, has established itself as one of the most widely utilized inhalation anesthetics in contemporary clinical practice across diverse patient populations and surgical settings (Edgington et al., 2023; Morse and Anderson, 2025). Approved by the FDA for induction and maintenance of general anesthesia in both adult and pediatric patients, sevoflurane provides hypnosis, amnesia, analgesia, akinesia, and autonomic blockade during surgical and procedural interventions (Edgington et al., 2023). Its clinical prominence stems from favorable pharmacokinetic properties, including a low blood-gas partition coefficient facilitating rapid induction and emergence, minimal airway irritation enabling smooth mask induction, and a pleasant odor that enhances patient acceptance, particularly in pediatric populations (Morse and Anderson, 2025; Smith et al., 1996). At the molecular level, sevoflurane exerts its anesthetic effects through modulation of multiple neurotransmitter systems, including potentiation of gamma-aminobutyric acid (GABA) inhibition and inhibition of glutamatergic NMDA receptor activity (Mapelli et al., 2021).

Despite sevoflurane’s widespread clinical application and generally favorable safety profile, accumulating evidence suggests potential for CNS complications, particularly in the context of polypharmacy inherent to modern anesthetic practice. The complexity of perioperative medication management necessitates careful consideration of potential drug interactions. In the perioperative period, patients receive multiple drugs that may interact with each other, potentially affecting treatment efficacy and safety through pharmacodynamic, pharmacokinetic, and pharmaceutical interactions (Silva et al., 2023). When patients are taking multiple medications, perioperative clinicians must make complex decisions regarding medication continuation or discontinuation, as inappropriate management may lead to adverse events or surgical cancellation (Sahai et al., 2022). This complexity is particularly pronounced in anesthesia practice, where sevoflurane is invariably combined with other CNS-active agents including opioid analgesics, benzodiazepines, intravenous anesthetics, neuromuscular blocking agents, and antiemetics to achieve the multifaceted goals of modern balanced anesthesia (Silva et al., 2023; Kianian et al., 2024).

Risk factors for opioid-related adverse drug events in surgical patients include advanced age, specific comorbidities, use of benzodiazepines or gabapentinoids, and higher opioid doses (Yiu et al., 2022). Perioperative medication administration poses considerable safety concerns, with many errors being detected only after causing significant physiological disturbances, while the intricacy of medication administration in the perioperative setting presents specific challenges to patient safety (Ye, 2023). While anesthesiology has been a leader in medicine for patient safety research and implementing standards of care, improved anesthesia patient safety has not been uniformly obtained worldwide, with differences often related to factors such as availability of healthcare resources, trained personnel, and safety data collection efforts (Warner et al., 2022).

Spontaneous adverse event reporting systems, particularly the FAERS, have emerged as valuable tools for post-marketing pharmacovigilance, enabling detection of rare adverse drug reactions and DDIs that may not be apparent in pre-approval clinical trials due to limited sample sizes and exclusion of polypharmacy scenarios (Fusaroli et al., 2024; Bate and Evans, 2009). Disproportionality analysis of FAERS data allows for systematic identification of statistical signals representing unexpected associations between drugs or drug combinations and specific adverse events, thereby generating hypotheses warranting further investigation (Montastruc et al., 2011; Raschi et al., 2022). It is important to recognize that FAERS records reported events rather than confirmed causal adverse drug reactions, and that disproportionality signals reflect reporting patterns rather than true incidence or established risk (Noguchi et al., 2021a). Appropriate interpretation therefore requires methodological rigor in both analysis and inference.

Despite the clinical importance of understanding sevoflurane’s safety profile in the context of polypharmacy, a significant knowledge gap exists regarding specific DDI-related CNS adverse events. While isolated case reports have documented movement disorders, emergence delirium, and other neurological complications associated with sevoflurane-containing anesthetic regimens, no comprehensive pharmacovigilance study has systematically evaluated the spectrum and magnitude of CNS adverse event signals associated with sevoflurane drug combinations using large-scale real-world data. The present study addresses this critical gap by leveraging the FAERS database to identify disproportionality signals for CNS adverse events associated with sevoflurane DDIs, with particular focus on characterizing high-risk drug combinations and vulnerable patient populations. To ensure analytical robustness, we adopted a dual-framework approach combining a specificity-focused PS-restricted primary analysis with a sensitivity-focused role-independent complementary analysis, complemented by vulnerable population subgroup analyses and time-to-event characterization. To address the known limitations of frequency-based interaction measures, the Ω shrinkage measure was applied as a Bayesian validation method for all interaction signals, enabling distinction between true synergistic signals and potential false positives (Noguchi et al., 2020). Through application of advanced statistical methodologies including interaction signal quantification, this investigation aims to provide hypothesis-generating evidence to inform clinical decision-making and guide future prospective validation studies.

## Materials and methods

### Data source

This pharmacovigilance study utilized the publicly available FDA Adverse Event Reporting System (FAERS) database. FAERS is a spontaneous reporting system containing adverse event reports submitted by healthcare professionals, consumers, and pharmaceutical manufacturers. As a spontaneous reporting database, FAERS records reported events that may or may not represent confirmed causal adverse drug reactions; reporter-assigned drug roles reflect clinical judgment rather than formal causality categories such as those defined by the WHO-UMC framework. All reports from the first quarter of 2004 (when FAERS data format was standardized) through the second quarter of 2025 were extracted. The database consists of seven data files: DEMO (demographics and administrative information), DRUG (drug information), REAC (adverse event terms), OUTC (patient outcomes), RPSR (report sources), THER (drug therapy dates), and INDI (indications for use). Adverse events are coded using the Medical Dictionary for Regulatory Activities (MedDRA) terminology.

### Data processing and deduplication

Data cleaning followed FDA-recommended protocols. For duplicate reports sharing identical CASEID, only the report with the most recent FDA_DT (FDA receipt date) was retained. When both CASEID and FDA_DT were identical, the report with the highest PRIMARYID was selected. This deduplication process minimized case count inflation. Only reports containing at least one drug record and at least one adverse event record were included in the analytical dataset.

### Study cohorts

Sevoflurane was identified by searching “SEVOFLURANE” and trade names (“ULTANE”, “SEVORANE”) in the DRUGNAME and PROD_AI fields. Two parallel analytical cohorts were constructed to address the inherent tension between specificity and sensitivity in DDI signal detection.

The PS-restricted primary cohort included reports listing sevoflurane as Primary Suspect (PS) drug, indicating the reporter’s suspected causal relationship. Within this cohort, two sub-cohorts were established: the concomitant use cohort included PS sevoflurane reports with at least one other concomitant medication; the quasi-monotherapy reference cohort included PS sevoflurane reports with either no other drugs or only drugs unlikely to cause CNS effects (intravenous fluids, electrolytes). This reference cohort served to isolate additional signal contributions from concomitant medications.

The role-independent complementary cohort included all reports listing sevoflurane in any role designation (PS, Secondary Suspect, Concomitant, or Interacting), reflecting the principle that interaction signals are distributed across multiple role combinations rather than concentrated solely under PS attribution. This cohort was used to compute role-independent disproportionality and interaction estimates as a complement to the PS-restricted primary analysis.

### Outcome definition

Target adverse events comprised all reports classified under “Nervous system disorders” System Organ Class (SOC) in MedDRA. Throughout this study, “adverse events” refers to reported events as captured in FAERS, not adjudicated adverse drug reactions. A predefined list of CNS adverse events of clinical interest was established (Table 1), including seizure-related events (seizure, status epilepticus, convulsion, epilepsy), consciousness disorders (coma, stupor, sedation, somnolence, lethargy), cognitive disorders (cognitive disorder, amnesia, mental impairment), movement disorders (dyskinesia, dystonia, hypertonia, tremor), encephalopathy, and delirium-related states (delirium, confusional state, agitation, hallucination). Adverse events were analyzed at the Preferred Term (PT) level.

**TABLE 1:** Predefined central nervous system adverse events of interest based on MedDRA hierarchy.

System organ class (SOC)	High level term (HLT)	Preferred terms (PTs) of interest
Nervous system disorders	Seizures and seizure disorders NEC	Seizure, status epilepticus, convulsion, epilepsy
Nervous system disorders	Disturbances in consciousness NEC	Coma, stupor, sedation, somnolence, lethargy
Nervous system disorders	Cognitive and attention disorders	Cognitive disorder, amnesia, mental impairment
Nervous system disorders	Movement disorders and dyskinesias NEC	Dyskinesia, dystonia, hypertonia, tremor
Nervous system disorders	Encephalopathies	Encephalopathy
Psychiatric disorders	Delirium states	Delirium, confusional state, agitation, hallucination

Abbreviations: SOC, system organ class; HLT, high level term; PT, preferred term; MedDRA, medical dictionary for regulatory activities; NEC, Not Elsewhere Classified. Adverse events were analyzed at the PT, level throughout this study.

### Signal detection methods

Disproportionality analysis was performed to identify drug-event combinations with reporting frequencies exceeding expected background rates. Three complementary approaches were employed: Reporting Odds Ratio (ROR) as the primary measure, Proportional Reporting Ratio (PRR) for confirmatory analysis, and two Bayesian methods for supplementary multi-method validation.

Signal calculations used standard 2 × 2 contingency tables: cell “a” (target event with target drug combination), cell “b” (other events with target drug combination), cell “c” (target event with other drugs), and cell “d” (other events with other drugs).

ROR was calculated as (a/c)/(b/d), with positive signals defined when the lower 95% confidence interval (CI) limit exceeded 1 and cell “a” ≥3. PRR was calculated as [a/(a+b)]/[c/(c + d)], with positive signals defined when PRR ≥2, chi-square (χ^2^) ≥4, and cell “a” ≥3.

The Empirical Bayesian Geometric Mean (EBGM) was calculated using the Gamma-Poisson Shrinker model, which applies Bayesian shrinkage toward the null, inherently penalizing estimates from small counts. Signals were identified when EB05 (lower 5th percentile of the posterior distribution) exceeded 1. The Information Component (IC) was calculated using the Bayesian confidence propagation neural network framework, with signals identified when IC025 (lower 2.5th percentile of the posterior) exceeded 0.

### Analytical strategy

**FIGURE 1:**
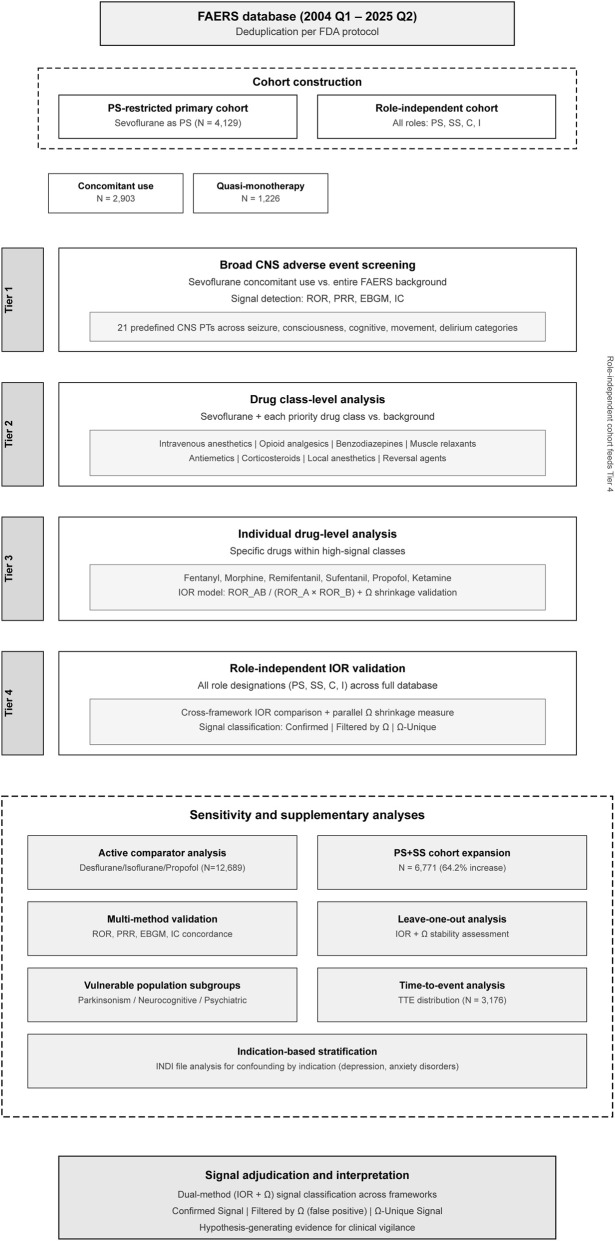
The study flowchart.

The overall analytical workflow is summarized in Figure 1. A four-tier hierarchical approach was implemented, progressing from broad screening to focused investigation and finally to role-independent validation. The first tier conducted broad screening by calculating signals for all predefined CNS adverse events comparing sevoflurane concomitant use against the entire database background. The second tier performed drug class-level analysis, evaluating signals for sevoflurane combinations with priority drug classes including intravenous anesthetics, opioid analgesics, benzodiazepines, non-depolarizing muscle relaxants, antiemetics, corticosteroids, local anesthetics, and reversal agents. These drug classes were selected based on their frequent concomitant use with sevoflurane, known CNS pharmacodynamic interactions, and clinical relevance in anesthesia practice. The third tier involved individual drug-level analysis, examining specific drugs within classes demonstrating strong signals in the second-tier analysis. The fourth tier performed role-independent analysis using the complementary cohort to evaluate interaction signals across all role combinations, addressing the probabilistic nature of co-reporting in spontaneous reporting systems. For all key signals identified, stratified analyses by age groups (pediatric <18 years, adult 18–65 years, elderly >65 years) and sex were conducted to identify potentially vulnerable populations.

### Interaction signal analysis

To distinguish synergistic interactions from additive effects, an Interaction Reporting Odds Ratio (IOR) model was applied. This method quantifies whether observed combination signals exceed expected additive effects.

The IOR model required three independent calculations: ROR_AB (sevoflurane + Drug X versus all others), ROR_A (sevoflurane quasi-monotherapy versus all others), and ROR_B (Drug X alone versus all others). The IOR was computed as ROR_AB/(ROR_A × ROR_B), with supra-additive (synergistic) interaction identified when the lower bound of the 95% CI exceeded 1. This ratio-of-ratios formulation is inherently agnostic to drug role attribution, as it independently quantifies each drug’s baseline disproportionality before evaluating combination effects.

To address the known sensitivity of frequency-based IOR to sparse data and reporting variability, the Ω (Omega) shrinkage measure was applied as a Bayesian validation method for all IOR signals (Noguchi et al., 2020). Unlike the IOR, which is derived from observed reporting frequencies, the Ω shrinkage measure employs a Bayesian framework specifically designed for DDI signal detection in spontaneous reporting systems, applying conservative shrinkage that penalizes estimates from small sample sizes and reduces false-positive rates. A signal was considered validated when the lower bound of the 95% CI of the Ω estimate exceeded 0. The combined application of IOR and Ω enabled classification of signals as “Confirmed Signal” (positive by both methods), “Filtered by Ω” (IOR-positive but Ω-negative, suggesting a potential false positive), or “Ω-Unique Signal” (IOR-negative but Ω-positive, indicating signals missed by frequency thresholding).

This interaction analysis focused on combinations with opioid analgesics, benzodiazepines, and intravenous anesthetics that demonstrated the strongest disproportionality signals. Identical IOR and Ω computations were performed in parallel using both the PS-restricted cohort and the role-independent complementary cohort to assess signal robustness across role attribution frameworks. Identified IOR signals were classified by signal strength: strong (IOR lower 95% CI > 1 and N ≥ 10), moderate (IOR lower 95% CI > 1 and 5 ≤ N < 10), and exploratory (IOR lower 95% CI > 1 and 3 ≤ N < 5).

### Statistical analysis

All analyses were performed using R software (version 4.3.2). ROR and PRR values were reported with 95% CIs. Chi-square tests assessed statistical significance for PRR calculations. The Ω shrinkage measure was computed using the approach described by Noguchi et al. (Noguchi et al., 2020), with 95% CIs derived from the posterior distribution. Descriptive statistics included frequencies and percentages for categorical variables and means with standard deviations for continuous variables. Statistical significance was set at two-sided alpha = 0.05.

### Sensitivity analysis

Multiple sensitivity analyses were performed to assess the robustness of the primary findings. To address potential confounding by indication, sensitivity analyses utilized the INDI file to stratify cases reporting specific indications (e.g., depression, anxiety disorders) that might independently influence delirium risk. To evaluate the impact of PS-only restriction, an expanded cohort including both PS and secondary suspect (SS) designations was analyzed and compared with the primary results. Additionally, a fully role-independent analysis incorporating all four role designations (PS, SS, C, I) was conducted to provide complete role-coverage validation, as interaction signals in spontaneous reporting systems may distribute across PS-PS, PS-NonPS, NonPS-PS, and NonPS-NonPS combinations.

All IOR signals in both the PS-restricted and role-independent frameworks were validated using the Ω shrinkage measure to distinguish confirmed interaction signals from potential false positives. Multi-method concordance across ROR, EBGM, and IC was evaluated for all key drug-event pairs. Leave-one-out analyses were performed for the statistically significant IOR signals with parallel Ω validation to assess sensitivity to sequential case removal. The role-independent IOR analysis provided additional cross-framework validation complemented by Ω shrinkage assessment for the core signals, reinforcing the importance of role-coverage flexibility in DDI signal detection.

To address potential confounding by indication inherent in comparing sevoflurane against the full FAERS database background, an active comparator sensitivity analysis was performed. A comparator cohort (N = 12,689) was constructed comprising patients reporting desflurane, isoflurane, or propofol as monotherapy. Main effect disproportionality was calculated comparing sevoflurane against this active comparator background. DDI signals were re-evaluated within this restricted background using both IOR and Ω shrinkage measure. This approach mitigates confounding from the perioperative context, as comparator patients share similar surgical indications and healthcare settings.

Vulnerable Population Subgroup Analysis. To explore whether baseline neurological vulnerability amplifies the risk of CNS adverse events under sevoflurane exposure, a *post hoc* exploratory subgroup analysis was performed by jointly searching the INDI and REAC tables for keywords corresponding to three predefined vulnerable populations: parkinsonism, neurocognitive impairment, and psychiatric conditions. Within each subgroup, the reporting frequency of specific CNS adverse events was compared with that of non-vulnerable sevoflurane users using ROR. As vulnerable populations were identified via INDI-table keyword matching, these subgroups represent proxies rather than clinically confirmed diagnoses.

Time-to-Event Analysis. To characterize the temporal pattern of sevoflurane-related CNS events and evaluate the plausibility of acute pharmacodynamic interactions, time-to-event (TTE) was calculated as the difference between the adverse event date (EVENT_DT) and the drug start date (START_DT). Cases with valid date pairs were stratified into four intervals: 0 days (same day), 1–7 days, 8–30 days, and >30 days.

### Ethical considerations

This study analyzed publicly available, de-identified FAERS data. As the research involved secondary analysis of anonymized surveillance data without patient contact, institutional review board approval was not required per regulations governing publicly available de-identified datasets.

## Results

### Baseline characteristics of study cohorts

After implementing the rigorous deduplication process, a total of 4,129 reports listing sevoflurane as a primary suspect drug were identified in the FAERS database from the first quarter of 2004 through the second quarter of 2025. Of these, 2,903 reports (70.3%) were classified into the concomitant use cohort, while 1,226 reports (29.7%) comprised the quasi-monotherapy reference cohort.

The baseline characteristics of both cohorts are presented in Table 2. The concomitant use cohort demonstrated a higher mean age compared to the quasi-monotherapy cohort (37.0 ± 25.8 years versus 28.6 ± 24.9 years). Regarding age distribution, pediatric patients (<18 years) represented similar proportions in both cohorts (17.7% versus 17.9%), whereas adult patients (18–65 years) were more prevalent in the concomitant use cohort (26.7% versus 16.0%), and elderly patients (>65 years) showed a similar pattern (9.9% versus 4.8%).

**TABLE 2:** Baseline characteristics of study cohorts.

Characteristic	Concomitant use (N = 2903)	Quasi-monotherapy (N = 1226)
Age years - mean (SD)	37.0 (25.8)	28.6 (24.9)
Pediatric (<18 years) n (%)	513 (17.7%)	219 (17.9%)
Adult (18–65 years) n (%)	776 (26.7%)	196 (16.0%)
Elderly (>65 years) n (%)	288 (9.9%)	59 (4.8%)
Female n (%)	1002 (34.5%)	278 (22.7%)
Male n (%)	988 (34.0%)	320 (26.1%)
Death n (%)	290 (10.0%)	162 (13.2%)
Hospitalization n (%)	794 (27.4%)	220 (17.9%)
Life-threatening n (%)	484 (16.7%)	102 (8.3%)

Abbreviations: SD, Standard Deviation. Data source: FDA, Adverse Event Reporting System (FAERS), 2004Q1–2025Q2. Percentages may not sum to 100% due to missing data in age, sex, or outcome fields. Concomitant use cohort: sevoflurane as PS, with ≥ 1 concomitant medication; Quasi-monotherapy cohort: sevoflurane as PS, with no other drugs or only non-CNS-active agents.

Sex distribution revealed that females constituted 34.5% of the concomitant use cohort and 22.7% of the quasi-monotherapy cohort, while males comprised 34.0% and 26.1%, respectively. Notably, serious outcomes were frequently reported in both cohorts. Death was reported in 10.0% of concomitant use cases versus 13.2% of quasi-monotherapy cases. Hospitalization was documented in 27.4% versus 17.9%, and life-threatening events in 16.7% versus 8.3% of cases, respectively.

### First-tier analysis: overall CNS adverse event signals

The broad screening analysis comparing all sevoflurane concomitant use reports against the database background identified multiple significant disproportionality signals for specific CNS adverse events (Table 3). Among the predefined CNS adverse events of interest, several demonstrated statistically significant signals by both ROR and PRR criteria.

**TABLE 3:** First-tier disproportionality analysis: CNS adverse event signals for sevoflurane concomitant use.

Adverse event	Cases (n)	ROR (95% CI)	PRR (95% CI)	Chi-square	Signal status
Dystonia	26	8.94 (6.10–13.11)	8.87 (6.05–13.00)	169.66	Positive
Hypertonia	9	10.12 (5.35–19.14)	10.09 (5.33–19.08)	60.63	Positive
Status epilepticus	8	5.19 (2.65–10.17)	5.18 (2.64–10.15)	20.90	Positive
Dyskinesia	24	4.23 (2.84–6.30)	4.21 (2.83–6.26)	53.74	Positive
Agitation	38	3.55 (2.59–4.89)	3.52 (2.56–4.84)	64.79	Positive
Delirium	17	3.47 (2.17–5.55)	3.45 (2.16–5.53)	25.85	Positive
Coma	20	2.87 (1.86–4.42)	2.85 (1.85–4.40)	21.15	Positive
Convulsion	25	2.27 (1.53–3.35)	2.25 (1.53–3.33)	15.44	Positive
Confusional state	21	0.91 (0.59–1.39)	0.91 (0.59–1.39)	0.20	Negative
Somnolence	21	0.75 (0.49–1.15)	0.76 (0.49–1.16)	1.70	Negative
Seizure	11	0.79 (0.44–1.41)	0.79 (0.44–1.41)	0.64	Negative
Cognitive disorder	9	1.43 (0.76–2.70)	1.43 (0.76–2.70)	0.52	Negative
Tremor	7	0.31 (0.15–0.63)	0.31 (0.15–0.64)	11.52	Negative
Sedation	6	1.80 (0.84–3.89)	1.80 (0.83–3.89)	1.00	Negative
Hallucination	5	0.52 (0.22–1.19)	0.52 (0.22–1.20)	2.47	Negative
Lethargy	5	0.66 (0.28–1.52)	0.66 (0.28–1.52)	0.98	Negative
Epilepsy	4	1.01 (0.40–2.55)	1.01 (0.40–2.55)	0.00	Negative
Encephalopathy	3	0.96 (0.34–2.75)	0.96 (0.34–2.75)	0.00	Negative
Amnesia	3	0.35 (0.12–1.01)	0.35 (0.12–1.01)	4.15	Negative
Mental impairment	2	0.70 (0.20–2.42)	0.70 (0.20–2.42)	0.32	Negative
Stupor	1	3.04 (0.61–15.07)	3.04 (0.61–15.06)	0.00	Negative

Abbreviations: ROR, reporting odds ratio; PRR, proportional reporting ratio; CI, Confidence Interval. Data source: FDA, Adverse Event Reporting System (FAERS), 2004Q1–2025Q2. Signal criteria: ROR, positive when lower 95% CI > 1 and N ≥ 3; PRR, positive when PRR ≥2, χ^2^ ≥ 4, and N ≥ 3. “Positive” indicates both criteria met; “Negative” indicates one or both criteria not met.

The strongest signals were observed for movement disorders. Hypertonia exhibited the highest signal intensity with an ROR of 10.12 (95% CI: 5.35–19.14) and PRR of 10.09 based on 9 cases. Dystonia showed an ROR of 8.94 (95% CI: 6.10–13.11) and PRR of 8.87 with 26 reported cases. Status epilepticus demonstrated an ROR of 5.19 (95% CI: 2.65–10.17) and PRR of 5.18 with 8 cases. Dyskinesia exhibited an ROR of 4.23 (95% CI: 2.84–6.30) and PRR of 4.21 based on 24 cases.

For consciousness and delirium-related disorders, significant signals were detected for agitation (ROR: 3.55, 95% CI: 2.59–4.89; 38 cases), delirium (ROR: 3.47, 95% CI: 2.17–5.55; 17 cases), and coma (ROR: 2.87, 95% CI: 1.86–4.42; 20 cases). Convulsion showed an ROR of 2.27 (95% CI: 1.53–3.35) with 25 cases.

Conversely, several CNS adverse events did not meet signal criteria. These included confusional state (ROR: 0.91, 95% CI: 0.59–1.39), somnolence (ROR: 0.75, 95% CI: 0.49–1.15), seizure (ROR: 0.79, 95% CI: 0.44–1.41), tremor (ROR: 0.31, 95% CI: 0.15–0.63), hallucination (ROR: 0.52, 95% CI: 0.22–1.19), and amnesia (ROR: 0.35, 95% CI: 0.12–1.01).

### Second-tier analysis: drug class-level signal distribution

Analysis of signal frequencies across priority drug classes revealed differential patterns of CNS adverse event reporting (Table 4). Intravenous anesthetics and opioid analgesics demonstrated the highest signal rates, each yielding 8 positive signals by both ROR and PRR criteria out of 18 total drug class-event combinations analyzed, representing a signal rate of 44.4%. Corticosteroids showed a signal rate of 42.9% (3 signals from 7 combinations), followed by antiemetics at 33.3% (3 from 9 combinations). Benzodiazepines and non-depolarizing muscle relaxants both demonstrated signal rates of 25.0% (4 from 16 combinations each for benzodiazepines, considering both ROR and PRR concordance). Local anesthetics exhibited a signal rate of 18.2% (2 from 11 combinations), while reversal agents showed the lowest rate at 14.3% (1 from 7 combinations).

**TABLE 4:** Signal distribution across priority drug classes.

Drug class	Total combinations evaluated	ROR signals	PRR signals	Both criteria met	Signal rate (%)
Intravenous anesthetics	18	8	8	8	44.4
Opioid analgesics	18	8	8	8	44.4
Corticosteroids	7	3	3	3	42.9
Antiemetics	9	3	3	3	33.3
Benzodiazepines	16	4	4	4	25.0
Non-depolarizing muscle relaxants	16	6	4	4	25.0
Local anesthetics	11	2	2	2	18.2
Reversal agents	7	1	1	1	14.3

Signal Rate (%) = number of drug class–event combinations meeting both ROR, and PRR, signal criteria/total combinations evaluated × 100. Data source: FDA, Adverse Event Reporting System (FAERS), 2004Q1–2025Q2. Signal criteria: ROR, lower 95% CI > 1 and N ≥ 3; PRR ≥2, χ^2^ ≥ 4, and N ≥ 3.

Detailed drug class-specific signals are presented in Table 5. For antiemetics, the most prominent signal was dystonia with an exceptionally high ROR of 47.85 (95% CI: 21.77–105.20) based on 6 cases. Additional signals included dyskinesia (ROR: 12.91, 95% CI: 4.47–37.32; 3 cases) and seizure (ROR: 5.16, 95% CI: 1.79–14.91; 3 cases).

**TABLE 5:** Second-tier disproportionality analysis: CNS adverse event signals by drug class.

Drug class	Adverse event	Cases (n)	ROR (95% CI)	PRR (95% CI)	Chi-square
Corticosteroids	Dystonia	10	63.35 (33.92–118.29)	59.52 (31.87–111.14)	496.05
​	Dyskinesia	5	16.49 (7.05–38.57)	16.00 (6.84–37.41)	50.68
​	Somnolence	5	3.35 (1.43–7.84)	3.28 (1.40–7.67)	4.86
Antiemetics	Dystonia	6	47.85 (21.77–105.20)	45.65 (20.77–100.34)	203.28
​	Dyskinesia	3	12.91 (4.47–37.32)	12.61 (4.36–36.45)	18.0
​	Seizure	3	5.16 (1.79–14.91)	5.05 (1.75–14.60)	4.8
Benzodiazepines	Agitation	29	15.38 (10.61–22.29)	14.59 (10.06–21.14)	348.57
​	Dyskinesia	9	8.96 (4.71–17.01)	8.82 (4.64–16.75)	51.34
​	Coma	6	4.94 (2.28–10.70)	4.89 (2.26–10.60)	13.15
​	Convulsion	6	3.13 (1.45–6.79)	3.11 (1.43–6.73)	5.59
Intravenous anesthetics	Dystonia	22	15.62 (10.30–23.70)	15.39 (10.14–23.34)	274.83
​	Status epilepticus	6	8.12 (3.76–17.55)	8.09 (3.74–17.48)	27.47
​	Agitation	28	5.42 (3.74–7.85)	5.33 (3.68–7.72)	92.1
​	Dyskinesia	11	4.06 (2.27–7.25)	4.03 (2.26–7.21)	20.58
​	Delirium	8	3.44 (1.75–6.76)	3.43 (1.75–6.73)	10.19
​	Convulsion	14	2.64 (1.57–4.42)	2.62 (1.56–4.39)	11.52
Opioid analgesics	Dystonia	21	12.98 (8.48–19.87)	12.82 (8.38–19.63)	211.48
​	Status epilepticus	7	8.17 (3.99–16.75)	8.14 (3.97–16.68)	33.8
​	Delirium	13	4.78 (2.80–8.17)	4.75 (2.78–8.11)	32.88
​	Dyskinesia	15	4.78 (2.90–7.88)	4.74 (2.88–7.82)	38.69
​	Coma	12	3.12 (1.79–5.44)	3.10 (1.78–5.41)	13.87
​	Convulsion	19	3.10 (1.98–4.84)	3.07 (1.97–4.80)	23.38
Local anesthetics	Hypertonia	3	39.34 (13.70–112.93)	38.85 (13.53–111.54)	64.72
​	Dyskinesia	4	8.20 (3.23–20.81)	8.08 (3.18–20.51)	15.66
Non-depolarizing muscle relaxants	Status epilepticus	7	12.94 (6.31–26.54)	12.85 (6.26–26.36)	60.04
​	Hypertonia	4	13.49 (5.34–34.06)	13.44 (5.32–33.93)	29.94
​	Dyskinesia	6	3.15 (1.46–6.82)	3.14 (1.45–6.79)	5.7
​	Coma	7	2.95 (1.44–6.05)	2.94 (1.43–6.02)	6.12
Reversal agents	Delirium	3	18.24 (6.29–52.89)	17.71 (6.11–51.34)	27.15

Abbreviations: ROR, reporting odds ratio; PRR, proportional reporting ratio; CI, Confidence Interval. Data source: FDA, Adverse Event Reporting System (FAERS), 2004Q1–2025Q2. Signal criteria: ROR, lower 95% CI > 1 and N ≥ 3; PRR ≥2, χ^2^ ≥ 4, and N ≥ 3. Only drug class–event combinations meeting both criteria are shown.

Benzodiazepine combinations demonstrated particularly strong signals for agitation, with an ROR of 15.38 (95% CI: 10.61–22.29) based on 29 cases, representing the highest case count among all drug class-event combinations. Other significant signals included dyskinesia (ROR: 8.96, 95% CI: 4.71–17.01; 9 cases), coma (ROR: 4.94, 95% CI: 2.28–10.70; 6 cases), and convulsion (ROR: 3.13, 95% CI: 1.45–6.79; 6 cases).

Corticosteroid combinations exhibited the highest magnitude ROR for dystonia at 63.35 (95% CI: 33.92–118.29) with 10 cases, along with signals for dyskinesia (ROR: 16.49, 95% CI: 7.05–38.57; 5 cases) and somnolence (ROR: 3.35, 95% CI: 1.43–7.84; 5 cases).

Intravenous anesthetics generated multiple significant signals across diverse CNS adverse events. The strongest signal was observed for dystonia (ROR: 15.62, 95% CI: 10.30–23.70; 22 cases), followed by status epilepticus (ROR: 8.12, 95% CI: 3.76–17.55; 6 cases), agitation (ROR: 5.42, 95% CI: 3.74–7.85; 28 cases), dyskinesia (ROR: 4.06, 95% CI: 2.27–7.25; 11 cases), delirium (ROR: 3.44, 95% CI: 1.75–6.76; 8 cases), cognitive disorder (ROR: 2.62, 95% CI: 1.34–5.14; 8 cases), convulsion (ROR: 2.64, 95% CI: 1.57–4.42; 14 cases), and coma (ROR: 2.43, 95% CI: 1.24–4.76; 8 cases).

Local anesthetics showed signals primarily for movement disorders, including hypertonia (ROR: 39.34, 95% CI: 13.70–112.93; 3 cases) and dyskinesia (ROR: 8.20, 95% CI: 3.23–20.81; 4 cases).

Non-depolarizing muscle relaxants demonstrated signals for status epilepticus (ROR: 12.94, 95% CI: 6.31–26.54; 7 cases), hypertonia (ROR: 13.49, 95% CI: 5.34–34.06; 4 cases), dyskinesia (ROR: 3.15, 95% CI: 1.46–6.82; 6 cases), coma (ROR: 2.95, 95% CI: 1.44–6.05; 7 cases), cognitive disorder (ROR: 2.33, 95% CI: 1.01–5.39; 5 cases, signal by ROR only), and convulsion (ROR: 2.12, 95% CI: 1.08–4.17; 8 cases, signal by ROR only).

Opioid analgesics exhibited a broad spectrum of CNS signals. The most prominent was dystonia with an ROR of 12.98 (95% CI: 8.48–19.87) based on 21 cases. Additional signals included status epilepticus (ROR: 8.17, 95% CI: 3.99–16.75; 7 cases), delirium (ROR: 4.78, 95% CI: 2.80–8.17; 13 cases), dyskinesia (ROR: 4.78, 95% CI: 2.90–7.88; 15 cases), coma (ROR: 3.12, 95% CI: 1.79–5.44; 12 cases), convulsion (ROR: 3.10, 95% CI: 1.98–4.84; 19 cases), agitation (ROR: 2.21, 95% CI: 1.29–3.78; 13 cases), and hypertonia (ROR: 6.63, 95% CI: 2.32–18.93; 3 cases).

Reversal agents demonstrated a signal exclusively for delirium, with an ROR of 18.24 (95% CI: 6.29–52.89) based on 3 cases.

### Third-tier analysis: individual drug-level signals

Following the identification of strong signals at the drug class level, focused analysis of specific drugs within the highest-signal classes was conducted (Table 6). Among opioid analgesics, fentanyl demonstrated multiple significant signals. The strongest was for dystonia (ROR: 13.22, 95% CI: 7.57–23.11; 12 cases), followed by status epilepticus (ROR: 12.44, 95% CI: 5.75–26.91; 6 cases), delirium (ROR: 7.80, 95% CI: 4.46–13.62; 12 cases), dyskinesia (ROR: 6.23, 95% CI: 3.48–11.14; 11 cases), convulsion (ROR: 2.64, 95% CI: 1.39–5.00; 9 cases), and agitation (ROR: 2.73, 95% CI: 1.44–5.17; 9 cases). Notably, somnolence (ROR: 0.93, 95% CI: 0.47–1.83) and seizure (ROR: 1.18, 95% CI: 0.51–2.73) did not meet signal criteria.

**TABLE 6:** Third-tier disproportionality analysis: CNS adverse event signals for individual drugs.

Drug name	Adverse event	Cases (n)	ROR (95% CI)	PRR (95% CI)	Chi-square
Morphine	Dystonia	8	66.64 (33.25–133.54)	62.41 (31.14–125.07)	401.58
​	Agitation	4	9.32 (3.64–23.85)	9.03 (3.53–23.13)	18.34
​	Dyskinesia	3	13.52 (4.67–39.09)	13.19 (4.56–38.13)	19.03
Fentanyl	Dystonia	12	13.22 (7.57–23.11)	13.06 (7.47–22.82)	116.31
​	Status epilepticus	6	12.44 (5.75–26.91)	12.36 (5.71–26.74)	47.11
​	Delirium	12	7.80 (4.46–13.62)	7.70 (4.41–13.46)	60.3
​	Dyskinesia	11	6.23 (3.48–11.14)	6.16 (3.45–11.03)	40.09
​	Agitation	9	2.73 (1.44–5.17)	2.71 (1.43–5.14)	7.18
​	Convulsion	9	2.64 (1.39–5.00)	2.62 (1.38–4.97)	6.61
Remifentanil	Status epilepticus	6	23.61 (10.89–51.20)	23.31 (10.75–50.56)	98.0
​	Convulsion	8	4.49 (2.28–8.84)	4.43 (2.25–8.72)	16.32
Sufentanil	Coma	7	8.21 (3.98–16.91)	8.06 (3.91–16.61)	33.55
​	Confusional state	11	3.84 (2.14–6.91)	3.75 (2.09–6.75)	18.29
​	Cognitive disorder	3	4.09 (1.43–11.73)	4.07 (1.42–11.65)	3.14
Propofol	Dystonia	22	16.10 (10.62–24.43)	15.86 (10.45–24.06)	284.45
​	Status epilepticus	6	8.37 (3.87–18.09)	8.33 (3.86–18.02)	28.6
​	Agitation	27	5.38 (3.69–7.85)	5.30 (3.63–7.73)	87.84
​	Dyskinesia	11	4.18 (2.34–7.48)	4.16 (2.33–7.43)	21.68
​	Delirium	8	3.55 (1.81–6.96)	3.53 (1.80–6.93)	10.81
​	Convulsion	14	2.72 (1.62–4.56)	2.70 (1.61–4.53)	12.37
​	Cognitive disorder	8	2.70 (1.38–5.30)	2.69 (1.37–5.28)	5.99
​	Coma	8	2.50 (1.27–4.91)	2.49 (1.27–4.89)	4.91

Abbreviations: ROR, reporting odds ratio; PRR, proportional reporting ratio; CI, Confidence Interval. Data source: FDA, Adverse Event Reporting System (FAERS), 2004Q1–2025Q2. Signal criteria: ROR, lower 95% CI > 1 and N ≥ 3; PRR ≥2, χ^2^ ≥ 4, and N ≥ 3. Only drug–event combinations meeting both criteria are shown. Empty rows at the bottom of the original table have been removed.

Morphine exhibited particularly high magnitude signals despite smaller case counts, with dystonia showing an ROR of 66.64 (95% CI: 33.25–133.54) based on 8 cases, representing the highest ROR observed among individual drug-event combinations. Additional signals included dyskinesia (ROR: 13.52, 95% CI: 4.67–39.09; 3 cases) and agitation (ROR: 9.32, 95% CI: 3.64–23.85; 4 cases).

Among intravenous anesthetics, propofol generated the most extensive signal profile. Dystonia exhibited an ROR of 16.10 (95% CI: 10.62–24.43) with 22 cases, followed by status epilepticus (ROR: 8.37, 95% CI: 3.87–18.09; 6 cases), agitation (ROR: 5.38, 95% CI: 3.69–7.85; 27 cases), dyskinesia (ROR: 4.18, 95% CI: 2.34–7.48; 11 cases), delirium (ROR: 3.55, 95% CI: 1.81–6.96; 8 cases), convulsion (ROR: 2.72, 95% CI: 1.62–4.56; 14 cases), cognitive disorder (ROR: 2.70, 95% CI: 1.38–5.30; 8 cases), and coma (ROR: 2.50, 95% CI: 1.27–4.91; 8 cases). Confusional state (ROR: 1.38, 95% CI: 0.84–2.28), somnolence (ROR: 0.93, 95% CI: 0.53–1.61), and seizure (ROR: 1.09, 95% CI: 0.53–2.23) did not meet signal criteria.

Remifentanil demonstrated strong signals specifically for seizure-related events, including status epilepticus (ROR: 23.61, 95% CI: 10.89–51.20; 6 cases) and convulsion (ROR: 4.49, 95% CI: 2.28–8.84; 8 cases).

Sufentanil exhibited signals for consciousness disorders, including coma (ROR: 8.21, 95% CI: 3.98–16.91; 7 cases), cognitive disorder (ROR: 4.09, 95% CI: 1.43–11.73; 3 cases), confusional state (ROR: 3.84, 95% CI: 2.14–6.91; 11 cases), convulsion (ROR: 3.10, 95% CI: 1.22–7.85; 4 cases, signal by ROR only), and agitation (ROR: 3.21, 95% CI: 1.27–8.12; 4 cases, signal by ROR only).

### Interaction signal analysis (PS-restricted cohort)

Application of the Interaction Reporting Odds Ratio (IOR) model to identify synergistic drug-drug interactions revealed three statistically significant supra-additive signals in the PS-restricted primary cohort (Table 7, upper panel). These IOR signals were subsequently validated using the Ω shrinkage measure (Supplementary Table S1).

**TABLE 7:** Interaction signal analysis: Supra-additive drug-drug interactions identified by IOR model under dual-framework analysis.

Drug combination	Adverse event	Cases (n)	IOR (95% CI)	Signal strength	Clinical interpretation
Panel A. PS-restricted cohort (specificity-focused primary analysis)					
Morphine + sevoflurane	Dystonia	8	9.46 (3.57–25.08)	Moderate	Supra-additive interaction by IOR; not validated by Ω under PS-restricted framework; validated by Ω under role-independent and active comparator frameworks
Fentanyl + sevoflurane	Dystonia	12	2.96 (1.22–7.17)	Strong	Supra-additive interaction; confirmed by Ω shrinkage measure; IOR attenuated but Ω robust under role-independent framework
Propofol + sevoflurane	Confusional state	15	2.56 (1.02–6.39)	Strong	Borderline IOR but confirmed by Ω shrinkage measure; validated across PS-restricted, role-independent, and active comparator frameworks
Panel B. Role-independent cohort (sensitivity-focused complementary analysis)					
Alfentanil + sevoflurane	Amnesia	4	10.75 (2.86–40.37)	Exploratory	Strongest role-independent signal; small N warrants cautious interpretation
Morphine + sevoflurane	Status epilepticus	6	2.98 (1.24–7.15)	Moderate	Novel role-independent signal masked under PS restriction
Morphine + sevoflurane	Tremor	15	2.73 (1.54–4.86)	Strong	Role-independent signal; suggests under-recognized motor system phenotype
Sufentanil + sevoflurane	Confusional state	18	2.58 (1.45–4.59)	Strong	Role-independent signal; consistent with consciousness disturbance pattern
Remifentanil + sevoflurane	Tremor	13	2.40 (1.21–4.80)	Strong	Role-independent signal; supports opioid–tremor interaction phenotype
Morphine + sevoflurane	Dystonia	19	1.80 (1.09–2.97)	Strong	Cross-framework robust signal; significant in both PS-restricted and role-independent analyses
Propofol + sevoflurane	Tremor	36	1.76 (1.13–2.74)	Strong	Role-independent signal; expands propofol interaction phenotype beyond consciousness disorders

Abbreviations: IOR, interaction reporting odds ratio; CI, confidence interval; PS, primary suspect.

Panel A IOR, computed using PS-designated sevoflurane cases against background; Panel B IOR, computed using all role designations (PS, SS, C, I) against background.

Signal strength classification: Strong (IOR, lower 95% CI > 1 and N ≥ 10), Moderate (IOR, lower 95% CI > 1 and 5 ≤ N < 10), Exploratory (IOR, lower 95% CI > 1 and 3 ≤ N < 5).

Cross-framework robustness: Morphine–Dystonia is the only drug–event pair achieving statistical significance in both PS-restricted and role-independent IOR, analyses.

Data source: FDA, Adverse Event Reporting System (FAERS), 2004Q1–2025Q2.

Ω shrinkage validation: In Panel A, Fentanyl–Dystonia and Propofol–Confusional state were confirmed by the Ω shrinkage measure (Supplementary Table S3); Morphine–Dystonia was not validated by Ω under the PS-restricted framework but achieved validation under the role-independent framework (Supplementary Table S6) and active comparator framework (Supplementary Table S12).

The strongest IOR signal was observed for the combination of sevoflurane and morphine for dystonia, with an IOR of 9.46 (95% CI: 3.57–25.08) based on 8 cases. However, the Ω shrinkage measure did not validate this signal (Ω: 4.08, 95% CI: −0.03 to 8.19; lower CI < 0), suggesting that the high IOR magnitude may have been inflated by small sample size and morphine’s intrinsic propensity for dystonia reporting. This signal was classified as moderate strength (5 ≤ N < 10) by IOR criteria; although multi-method validation confirmed concordance across all three disproportionality methods (ROR, EBGM EB05 = 6.83, IC IC025 = 2.77; Supplementary Table S2), the lack of Ω validation indicates that this finding should be interpreted with caution as a potential false positive under the frequency-based framework (Supplementary Table S3).

The combination of sevoflurane and fentanyl demonstrated a supra-additive interaction signal for dystonia with an IOR of 2.96 (95% CI: 1.22–7.17) based on 12 cases, classified as strong signal strength (N ≥ 10) with full disproportionality concordance (EBGM EB05 = 5.01, IC IC025 = 2.30; Supplementary Table S2). This signal was confirmed by the Ω shrinkage measure (Ω: 4.63, 95% CI: 0.57–8.70; lower CI > 0), supporting it as a true synergistic interaction signal (Supplementary Table S3).

Sevoflurane combined with propofol exhibited an interaction signal for confusional state, with an IOR of 2.56 (95% CI: 1.02–6.39) based on 15 cases. Although meeting the case count threshold for strong classification (N ≥ 10), the borderline IOR lower 95% CI (1.02) and lack of disproportionality concordance (EB05 = 0.82, IC025 = −0.32; multi-method validation showed that neither Bayesian measure reached the signal threshold, consistent with the absence of this pair from the positive-signal entries in Supplementary Table S2) initially raised concerns regarding signal robustness. However, the Ω shrinkage measure validated this signal (Ω: 4.94, 95% CI: 0.90–8.99; lower CI > 0), indicating that despite the borderline IOR, the interaction represents a true synergistic signal detectable by the more conservative Bayesian DDI-specific method (Supplementary Table S3). This finding was also particularly notable given that confusional state did not demonstrate a significant disproportionality signal in the first-tier broad screening analysis, suggesting that the interaction-specific effect may represent a clinically important phenomenon masked in overall population-level analyses.

In summary, dual-method evaluation using IOR and Ω shrinkage reclassified the three PS-restricted interaction signals: fentanyl–dystonia and propofol–confusional state were confirmed as true synergistic signals, whereas morphine–dystonia was filtered by the Ω algorithm, warranting cautious interpretation under the PS-restricted framework (Supplementary Table S3).

### Fourth-tier analysis: role-independent IOR validation

To address the inherent limitation that interaction signals in spontaneous reporting systems may distribute across multiple role combinations rather than concentrating under PS attribution alone, a parallel role-independent IOR analysis was conducted using all four role designations (PS, SS, C, I). Descriptive analysis (Supplementary Table S4) confirmed that signals were dispersed across role combinations, with no cases observed in the PS–PS configuration and substantial proportions in the NonPS–NonPS configuration (e.g., 80.2% for fentanyl–dystonia, 69.0% for propofol–dystonia), empirically validating the necessity of role-independent analysis.

The role-independent IOR analysis (Table 7, lower panel; Supplementary Table S5) yielded seven significant supra-additive signals by IOR criteria: alfentanil–amnesia (IOR: 10.75; N = 4), morphine–status epilepticus (IOR: 2.98; N = 6), morphine–tremor (IOR: 2.73; N = 15), sufentanil–confusional state (IOR: 2.58; N = 18), remifentanil–tremor (IOR: 2.40; N = 13), morphine–dystonia (IOR: 1.80; N = 19), and propofol–tremor (IOR: 1.76; N = 36). Ω shrinkage validation of these role-independent IOR signals (Supplementary Table S5) confirmed four as robust: morphine–tremor (Ω: 4.77, lower CI > 0), sufentanil–confusional state (Ω: 5.21, lower CI > 0), morphine–dystonia (Ω: 5.26, lower CI > 0), and propofol–tremor (Ω: 6.14, lower CI > 0). The morphine–status epilepticus signal was filtered by Ω (lower CI < 0). Notably, the morphine–dystonia signal, which was not validated by Ω under the PS-restricted framework, achieved Ω validation under the role-independent framework with a larger sample size (N = 19 vs. N = 8), suggesting that the PS-restricted Ω non-validation reflected sample size limitations rather than absence of a true interaction (Supplementary Table S6). The Ω analysis additionally identified numerous Ω-unique signals not captured by IOR, including fentanyl–dystonia (Ω: 7.48, lower CI = 3.52; IOR: 1.04, non-significant) and propofol–confusional state (Ω: 6.80, lower CI = 2.85; IOR: 1.06, non-significant), confirming that these core interaction signals remain robust even when IOR is attenuated by high baseline disproportionality across all roles (Supplementary Table S5).

Conversely, the fentanyl–dystonia IOR was attenuated from 2.96 to 1.04 under role-independent analysis, reflecting signal dilution attributable to fentanyl’s high baseline disproportionality across all roles. However, the Ω shrinkage measure confirmed this signal with a strong Ω of 7.48 (lower CI = 3.52), demonstrating that the Bayesian DDI-specific method can recover true interaction signals masked by IOR dilution. This bidirectional pattern of signal emergence and attenuation across methods indicates that single-method analyses may yield biased estimates and that complementary analytical strategies enhance interaction signal detection.

### Vulnerable Population Subgroup Analysis

A *post hoc* subgroup analysis comparing vulnerable populations with non-vulnerable sevoflurane users (Supplementary Table S7) revealed elevated CNS event reporting among indication-defined proxies for baseline neurological compromise. Patients with parkinsonism showed markedly increased signals for dystonia (ROR: 12.20), dyskinesia (ROR: 10.38), and agitation (ROR: 10.70). The neurocognitive impairment subgroup exhibited an exceptionally high signal for confusional state (ROR: 32.79). The psychiatric subgroup demonstrated elevated signals for agitation (ROR: 3.82), confusional state (ROR: 4.44), and delirium (ROR: 2.80). These exploratory findings are consistent with a “double-hit” hypothesis in which baseline neurological vulnerability may lower the threshold for drug-induced CNS adverse events.

### Time-to-Event Analysis

Among 3,176 sevoflurane-related CNS event reports with valid drug start and event dates, 70.5% (N = 2,238) occurred on the same day as drug administration, with an additional 18.9% within 1–7 days, 4.3% within 8–30 days, and 6.3% beyond 30 days (Supplementary Table S8). The pronounced clustering at Day 0 is consistent with acute pharmacodynamic interaction during or immediately after anesthesia, supporting the biological plausibility of the identified interaction signals.

### Sensitivity analyses

A sensitivity analysis using an expanded PS + SS cohort (N = 6,771, a 64.2% increase over the PS-only cohort) demonstrated concordant signal status for 71.6% (48/67) of evaluated drug-event pairs (Supplementary Table S9). Key signals including fentanyl-dystonia (PS + SS: ROR = 17.86, N = 38) and morphine-dystonia (PS + SS: ROR = 32.15, N = 11) remained significant in the expanded cohort.

Multi-method validation identified 21 drug-event pairs with strong concordance across all three methods (ROR, EBGM, and IC; Supplementary Table S2). Leave-one-out analyses with parallel Ω validation demonstrated complementary stability profiles across the two methods (Supplementary Table S10). For morphine–dystonia, the IOR signal retained significance after removing up to 5 of 8 cases, whereas the Ω signal was non-significant at all case counts, consistent with the PS-restricted Ω non-validation noted above. For fentanyl–dystonia, the Ω signal demonstrated greater stability than IOR, retaining significance after removing up to 3 cases (vs. 1 case for IOR). For propofol–confusional state, the Ω signal retained significance after removing up to 6 cases, substantially outperforming the IOR which lost significance upon removal of a single case. These results demonstrate that Ω provides superior stability for signals with adequate sample size, while IOR retains sensitivity for small-count signals where Ω may be overly conservative. The role-independent IOR analysis with Ω validation provided additional cross-framework confirmation for key signals and identified multiple previously masked interaction signals (Supplementary Tables S5, S6), reinforcing the importance of role-coverage flexibility and multi-method validation in DDI signal detection.

The active comparator sensitivity analysis further strengthened the robustness of the core findings. When sevoflurane was compared against an active comparator background of desflurane, isoflurane, and propofol monotherapy patients (N = 12,689), five CNS adverse events retained significant disproportionality signals: dystonia (ROR: 3.14, 95% CI: 1.66–5.92), convulsion (ROR: 2.28), somnolence (ROR: 2.34), coma (ROR: 1.95), and delirium (ROR: 2.23) (Supplementary Table S11). At the DDI level, the active comparator IOR and Ω analysis identified 10 significant drug–event combinations (Supplementary Table S12). Notably, morphine–dystonia was confirmed by both IOR (40.49, 95% CI: 11.34–144.56) and Ω (2.90, lower CI = 0.23) within the active comparator framework, providing additional evidence supporting this interaction signal despite its non-validation by Ω in the PS-restricted full-database analysis. Propofol combinations yielded multiple Ω-confirmed signals including agitation, dystonia, confusional state, convulsion, somnolence, and dyskinesia, consistent with the primary analysis findings.

## Discussion

This comprehensive pharmacovigilance analysis of the FAERS database identified substantial disproportionality signals for reported CNS adverse events associated with sevoflurane drug-drug interactions, particularly involving movement disorders and consciousness disturbances. Previous FAERS-based analysis of sevoflurane identified multiple adverse event signals across various system organ classes, yet the present study represents the first systematic investigation specifically focusing on DDI-related CNS complications. The findings revealed that intravenous anesthetics and opioid analgesics, each with signal rates of 44.4%, demonstrated the highest propensity for generating CNS adverse event signals when combined with sevoflurane. These findings suggest that these drug classes warrant particular clinical attention due to their potential pharmacodynamic interactions with sevoflurane on multiple neurotransmitter systems, which may contribute to compounded CNS effects.

An important methodological consideration in interpreting these findings is that FAERS records reported events rather than confirmed adverse drug reactions, and reporter-assigned drug roles reflect clinical judgment rather than formal causality categories. All disproportionality signals identified in this study represent statistical associations in reporting patterns and should be interpreted as hypothesis-generating findings rather than evidence of causal relationships (Noguchi et al., 2021a). To address this inherent property of spontaneous reporting systems, we adopted a dual-framework approach combining a specificity-focused PS-restricted primary analysis with a sensitivity-focused role-independent complementary analysis. The descriptive role distribution analysis confirmed that interaction signals are dispersed across multiple role combinations rather than concentrating under PS attribution, with substantial proportions of cases observed in the NonPS–NonPS configuration (e.g., 80.2% for fentanyl–dystonia, 69.0% for propofol–dystonia). Notably, no cases of any key drug–event pair were observed in the PS–PS combination, empirically validating the methodological necessity of role-independent analysis as a complement to PS-restricted disproportionality assessment (Fusaroli et al., 2024).

The predominance of movement disorder signals, particularly dystonia, warrants careful consideration. Consistent with the hierarchical analytical results, dystonia exhibited the strongest disproportionality signals across multiple drug classes in the second-tier analysis, with particularly high RORs observed for corticosteroid combinations and antiemetic combinations. Drug-induced dystonic reactions during anesthesia have been documented with various agents including propofol, sevoflurane, anti-emetics, antipsychotics and opioids, with the postulated mechanism involving an imbalance between dopaminergic and cholinergic neurotransmitters in the basal ganglia (Grötzsch et al., 2009). The notably high ROR for morphine-associated dystonia and the IOR signal identified through interaction analysis suggest a potential supra-additive reporting pattern that may reflect synergistic pharmacodynamic interactions. Under the PS-restricted framework, the morphine-dystonia IOR (9.46) was the highest among all identified signals; however, the Ω shrinkage measure did not validate this signal (lower CI < 0), indicating that the high IOR magnitude may have been inflated by small sample size. Under the role-independent framework with a larger sample (N = 19), both IOR (1.80) and Ω (5.26, lower CI = 1.24) confirmed this signal, suggesting that the PS-restricted non-validation reflected sample size constraints rather than absence of a true interaction pattern. Further supporting this signal, the active comparator analysis using desflurane, isoflurane, and propofol monotherapy patients as background confirmed the morphine-dystonia combination by both IOR and Ω (Supplementary Table S12). Although the morphine-dystonia PS-restricted IOR signal was based on 8 cases and classified as moderate strength under the predefined threshold framework, multi-method validation using Bayesian EBGM and IC methods confirmed this signal with strong concordance across all three methods, and leave-one-out analysis demonstrated IOR signal retention after removing up to 5 cases, supporting the disproportionality robustness of this finding. Acute dystonia has been reported during sevoflurane induction, with possible mechanisms involving altered dynamic relations between dopamine receptor blockade and other neurotransmitters (Bernard et al., 1999).

The role-independent analysis additionally identified seven supra-additive interaction signals by IOR criteria not captured by the PS-restricted framework, including alfentanil-amnesia, morphine-status epilepticus, morphine-tremor, sufentanil-confusional state, remifentanil-tremor, morphine-dystonia, and propofol-tremor. Ω shrinkage validation confirmed four of these seven signals (morphine-tremor, sufentanil-confusional state, morphine-dystonia, and propofol-tremor), while filtering morphine-status epilepticus as a potential false positive (Supplementary Table S5). These signals represent interaction-specific phenomena that emerge only when role-restriction bias is removed. Conversely, the fentanyl-dystonia IOR was attenuated from 2.96 to 1.04 under the role-independent framework, reflecting signal dilution attributable to fentanyl’s high baseline disproportionality across all roles. Notably, the Ω shrinkage measure recovered this signal with a strong Ω of 7.48 (lower CI = 3.52), demonstrating the capacity of Bayesian shrinkage methods to detect true interaction signals that are masked by IOR dilution. This bidirectional pattern of signal emergence and attenuation illustrates that single-framework analyses may yield biased estimates, and that interaction signal detection benefits from complementary role-coverage strategies and multi-method validation.

The incorporation of the Ω shrinkage measure as a Bayesian validation method for DDI signal detection represents an important methodological enhancement of this study. The Ω shrinkage measure, originally proposed by Noguchi et al. (Noguchi et al., 2020) and validated through subsequent studies (Noguchi et al., 2021b; Jung and Jung, 2024), applies conservative Bayesian shrinkage specifically designed to reduce false-positive rates in DDI signal detection from spontaneous reporting systems. Compared with frequency-based IOR, which is sensitive to sparse data and may inflate estimates when case counts are low, the Ω shrinkage measure penalizes small-count signals more heavily and provides more stable estimates (Noguchi et al., 2020). In the present study, the dual application of IOR and Ω enabled systematic reclassification of signals: in the PS-restricted framework, the morphine-dystonia IOR signal was filtered by Ω (suggesting potential false-positive inflation), while the fentanyl-dystonia and propofol-confusional state signals were confirmed. This complementary approach aligns with recent recommendations that multiple detection methods should be used jointly rather than relying on any single algorithm (Jung and Jung, 2024).

Movement disorders represented the most prominent safety signal category in the first-tier analysis, with hypertonia and dystonia demonstrating the highest signal intensities among all predefined CNS adverse events. The observed DDI signals likely reflect complex interactions at multiple levels of the nervous system. Opioids inhibit breathing at multiple, highly distributed sites in both central and peripheral nervous systems, affecting rhythmogenesis, pattern formation, and neuromodulation (Ramirez et al., 2021). When combined with sevoflurane, which also modulates GABAergic and glutamatergic neurotransmission, the potential for compounded CNS depression becomes evident. Risk factors for postoperative opioid-induced respiratory depression include older age, male sex, opioid naivety, sleep-disordered breathing and heart failure, with the PRODIGY trial demonstrating a 46% incidence of at least one respiratory depression event in postoperative patients (Jansen and Dahan, 2024).

Consciousness disorders, including agitation, delirium, and coma, demonstrated significant signals in the first-tier analysis. The strong signals for propofol-sevoflurane combinations, particularly regarding consciousness disorders, may be explained by shared molecular mechanisms. At the individual drug level, propofol combined with sevoflurane generated signals for agitation, delirium, and coma, consistent with the broader drug class findings. Both sevoflurane and propofol induce coherent frontal alpha oscillations and slow oscillations in humans through GABA-A receptor potentiation, suggesting a shared molecular and systems-level mechanism for the anesthesia-induced unconscious state (Purdon et al., 2013). This mechanistic overlap may potentiate adverse effects when drugs are combined, particularly in vulnerable populations such as pediatric or elderly patients. The propofol-sevoflurane interaction signal for confusional state was confirmed by the Ω shrinkage measure under both the PS-restricted (Ω: 4.94, lower CI = 0.90) and role-independent (Ω: 6.80, lower CI = 2.85) frameworks, and was further validated in the active comparator analysis (Supplementary Table S12), providing convergent evidence across multiple analytical approaches despite its borderline IOR lower confidence interval.

Among antiemetic combinations, dystonia exhibited an exceptionally high signal in the second-tier analysis. The antiemetic-associated dystonia signals, particularly with ondansetron, reflect a well-documented phenomenon. Ondansetron-induced dystonic reactions are thought to be caused by an imbalance between inhibitory and excitatory neurotransmitters in the extrapyramidal system (Baigent and Morris, 2023). The combination with sevoflurane may exacerbate this imbalance through additional modulation of multiple neurotransmitter systems, including serotonergic pathways.

The *post hoc* subgroup analysis revealed that baseline neurological vulnerability substantially amplifies the reporting frequency of specific CNS adverse events under sevoflurane exposure (Supplementary Table S7), consistent with a “double-hit” hypothesis in which preexisting neurological compromise may lower the threshold for drug-induced CNS effects. Patients with parkinsonism exhibited markedly elevated signals for dystonia (ROR: 12.20) and dyskinesia (ROR: 10.38), consistent with the established dopaminergic–cholinergic imbalance hypothesis underlying drug-induced extrapyramidal reactions (Noguchi et al., 2021a). The neurocognitive impairment subgroup demonstrated an exceptionally high signal for confusional state (ROR: 32.79), providing clinical context for the propofol–confusional state interaction signal observed in the broader analysis. The psychiatric subgroup showed elevated reporting of agitation, delirium, and consciousness disturbances, suggesting that pharmacologic and neurochemical vulnerabilities may converge during perioperative polypharmacy. These exploratory findings suggest the potential value of individualized risk stratification and heightened vigilance when administering sevoflurane in patients with preexisting neurological or psychiatric conditions.

Time-to-event characterization further supports the biological plausibility of the identified interaction signals (Supplementary Table S8). Among 3,176 reports with valid date pairs, 70.5% of CNS events occurred on the same day as drug administration, with an additional 18.9% reported within the first week. This pronounced temporal clustering aligns with acute pharmacodynamic interaction occurring during or immediately after anesthesia, rather than delayed cumulative toxicity or unrelated remote effects. The temporal pattern is consistent with the rapid onset and offset kinetics of sevoflurane and the acute neurochemical mechanisms hypothesized for the observed movement and consciousness disorders, reinforcing the clinical relevance of the identified signals.

A pediatric study evaluating adverse drug events due to CNS depressant drugs found that the risk of fatal or life-threatening adverse events was significantly higher with CNS depressant drugs than other drugs (12% vs. 2%), with pediatric patients without surgery showing higher risks than those with surgery (Yamamoto et al., 2020). This finding contextualizes the present study’s observations regarding age-stratified vulnerability, suggesting that the clinical context of drug administration significantly influences risk profiles. Recent reviews of informatics-driven approaches in pharmacovigilance have highlighted that DDIs contribute to 6%–30% of adverse drug event occurrences, emphasizing the public health significance of DDI signal detection (Ibrahim et al., 2021). The present study’s findings contribute quantitative evidence to this body of knowledge by identifying specific high-risk combinations and their associated CNS manifestations. A feasibility study of DDI signal detection in VigiBase demonstrated that signals of adverse drug interactions can be detected through broad statistical screening of individual case reports, though signal assessment requires detailed information on temporal relationships between drugs and adverse events (Hult et al., 2020). The hierarchical analytical approach employed in the present study addresses these challenges by systematically progressing from broad screening to focused investigation of specific drug combinations, supplemented by role-independent validation, Ω shrinkage verification, vulnerable population stratification, and time-to-event characterization.

The interaction signal analysis revealed two Ω-confirmed supra-additive interactions in the PS-restricted cohort (fentanyl-dystonia and propofol-confusional state) and four Ω-confirmed supra-additive signals in the role-independent analysis, indicating that these combinations exhibit reporting patterns exceeding simple additive models. The application of dual IOR and Ω methodology represents a significant methodological advancement over traditional disproportionality analysis. Computational frameworks for adverse drug interaction detection using disproportionality analysis can identify high-risk drug combinations by accounting for both individual drug effects and synergistic interactions (Bangard et al., 2025). The cross-framework comparison demonstrates that morphine-dystonia achieved Ω validation under the role-independent and active comparator frameworks, providing convergent evidence for this signal despite its non-validation by Ω under the PS-restricted framework. Among the PS-restricted findings, the fentanyl-dystonia signal demonstrated a robust evidence profile, classified as strong signal strength with full disproportionality concordance and Ω confirmation, although its IOR attenuation under role-independent analysis was compensated by strong Ω validation (Ω: 7.48). The propofol-confusional state signal, initially considered borderline based on IOR alone, was confirmed by Ω across all analytical frameworks, establishing it as a robust interaction signal. The FAERS database serves as a vital resource for pharmacovigilance, facilitating identification of potential drug-related adverse events through post-marketing surveillance (Potter et al., 2025). The present study’s comprehensive approach, analyzing over 4,000 sevoflurane reports spanning two decades, provides statistical power to detect rare but clinically significant adverse event signals. The use of multiple disproportionality and interaction-specific measures (ROR, PRR, IOR, and Ω) enhances signal robustness and reduces false-positive findings.

Several inherent limitations of FAERS-based research warrant acknowledgment. First, FAERS captures reported events rather than confirmed adverse drug reactions, and reporter-assigned drug roles do not constitute formal causality assessment (Fusaroli et al., 2024). All signals identified in this study are therefore hypothesis-generating and should not be interpreted as establishing causal relationships. The PS-restricted primary analysis was therefore designed to maximize specificity, while the role-independent complementary analysis was implemented to maximize sensitivity by capturing signals distributed across all role combinations. The PS + SS sensitivity analysis demonstrated 71.6% signal concordance, and the role-independent analysis provided cross-framework validation for core signals, supporting the robustness of the dual-framework approach. Second, several IOR signals are based on small case counts. The incorporation of the Ω shrinkage measure partially addresses this concern by applying conservative Bayesian shrinkage that penalizes estimates from small samples, as demonstrated by the filtering of the PS-restricted morphine-dystonia IOR signal. Nevertheless, signals based on fewer than 10 cases should be interpreted as moderate or exploratory findings rather than established safety signals. Third, multiple biases inherent to spontaneous reporting systems may influence the observed signals. Reporting bias, including underreporting and selective reporting, may differentially affect drug combinations depending on clinical awareness and reporting practices. Notoriety bias may inflate signals for well-known drug-event associations (e.g., opioid-related movement disorders) while masking less recognized interactions. Temporal trends in reporting practices, prescribing patterns, and regulatory attention may introduce time-varying confounding. Differential reporting practices across drugs, particularly between newer and established agents, may also affect comparative signal magnitudes. These biases cannot be fully addressed through statistical methods alone and represent inherent limitations of pharmacovigilance signal detection (Noguchi et al., 2021a). Disproportionality analyses face challenges including concerns about redundant analyses that add little new scientific understanding. However, the present study addresses these concerns through its novel focus on DDI-specific signals and comprehensive methodological approach. Key limitations of FAERS data include underreporting, reporting bias, lack of denominator data preventing incidence calculation, and absence of medical verification for reported information (Potter et al., 2025). Confounding by indication represents a particularly relevant challenge in anesthesia-related pharmacovigilance. Patients receiving multiple anesthetic agents typically have more complex surgical procedures or underlying comorbidities, potentially increasing baseline risk for adverse events. The active comparator sensitivity analysis, which compared sevoflurane against desflurane, isoflurane, and propofol monotherapy patients sharing similar perioperative contexts, partially mitigates this concern. The persistence of significant signals including dystonia, convulsion, coma, somnolence, and delirium within this restricted comparator framework (Supplementary Table S11), and the confirmation of DDI signals including morphine-dystonia and multiple propofol combinations by both IOR and Ω within the active comparator background (Supplementary Table S12), provide additional reassurance that the observed signals are not solely attributable to confounding by indication. The sensitivity analysis utilizing indication data further addresses this limitation, though incomplete reporting of indications in FAERS limits comprehensive adjustment. Spontaneous reporting systems contain voluntarily submitted reports with valuable demographic and clinical information, though mandatory reporting elements are limited and other information is often lacking (An et al., 2025). Disproportionality analysis using FAERS is a widely used method in pharmacovigilance, with multiple algorithmic approaches including ROR, PRR, BCPNN, and MGPS available for signal detection (Hu et al., 2025). While the present study employed both frequentist (ROR, PRR) and Bayesian (EBGM, IC, Ω) methods to enhance signal reliability, the lack of a gold standard reference set for sevoflurane DDI-related CNS adverse events limits definitive validation of identified signals.

The identified signals have important implications for perioperative medication management. During the perioperative period, patients receive several drugs that may interact with each other and affect treatment efficacy and safety, necessitating thorough review of medication lists and vigilant monitoring to detect signs of drug interactions (Silva et al., 2023). Clinicians should exercise heightened vigilance when administering sevoflurane in combination with high-signal drugs, particularly opioids and propofol, especially in potentially vulnerable populations identified by indication-based proxies for parkinsonism, neurocognitive impairment, or psychiatric conditions, as well as pediatric and elderly patients. The Ω-confirmed IOR values for certain combinations, particularly fentanyl-associated and propofol-associated interactions, warrant prospective clinical investigation to establish causality and elucidate underlying mechanisms. Disproportionality analysis provides valuable insights into drug safety through identification of both well-known and previously underreported adverse drug reactions, with findings having important implications for pharmacovigilance strategies and clinical risk assessment (Ye et al., 2025). Future research should employ prospective cohort designs or nested case-control studies within large perioperative databases to validate these signals and quantify absolute risk. Effective signal detection in pharmacovigilance requires addressing challenges in data quality and integration, timely decision-making, global regulatory compliance, and technological advancement. The present study’s findings should inform ongoing efforts to develop real-time clinical decision support systems that alert providers to high-risk drug combinations during anesthetic planning and administration.

The FDA has issued boxed warnings regarding serious risks including profound sedation, respiratory depression, coma, and death associated with concomitant use of opioids and benzodiazepines or other CNS depressants (Food and Administration, 2016). The present study’s findings support extension of similar precautionary guidance to sevoflurane combinations, particularly with opioids and intravenous anesthetics. Regulatory agencies should consider these signals when evaluating the need for enhanced labeling or risk mitigation strategies.

Knowledge graphs and advanced computational approaches in pharmacovigilance offer potential for improved adverse drug reaction prediction through integration of multiple data sources and application of machine learning techniques (Hauben et al., 2024). Future research should integrate FAERS signals with electronic health record data, clinical trial databases, and mechanistic pharmacological knowledge to develop comprehensive risk prediction models for sevoflurane DDI-related adverse events. Signal detection methods using routinely collected healthcare data should employ standardized outcome definitions, transparent code lists, and reproducible analytical approaches to enhance comparability across studies (Coste et al., 2023). The present study’s predefined analytical plan and comprehensive reporting of methods and results contributes to this goal and provides a framework for future DDI-focused pharmacovigilance investigations. Mechanistic studies examining the neurophysiological effects of specific drug combinations identified as high-risk in the present analysis could elucidate underlying pathways and identify potential protective strategies. Recent research demonstrating sevoflurane’s effects on specific NMDA receptor subtypes and their role in various CNS phenomena suggests potential molecular targets for understanding adverse interaction mechanisms (Guo et al., 2024). Such investigations could inform development of safer anesthetic protocols and individualized risk assessment strategies.

## Conclusion

This large-scale pharmacovigilance analysis identified multiple disproportionality signals for reported CNS adverse events associated with sevoflurane drug combinations, with movement disorders representing the most prominent safety concern. The dual-framework approach combining PS-restricted and role-independent analyses with parallel Ω shrinkage validation confirmed the fentanyl-dystonia and propofol-confusional state signals as robust Ω-validated interactions under the PS-restricted framework, while the morphine-dystonia signal achieved Ω validation under the role-independent and active comparator frameworks. The identification of supra-additive interaction signals for specific drug pairs provides hypothesis-generating evidence that warrants prospective clinical validation to establish causal relationships. These findings suggest the potential importance of vigilant monitoring and individualized risk-benefit assessment when administering sevoflurane in combination with opioids, intravenous anesthetics, and other CNS-active medications, particularly in patients with indicators of preexisting neurological vulnerability. Enhanced awareness of these potential associations and incorporation of pharmacovigilance signals into clinical decision-making processes may contribute to improved perioperative safety and patient outcomes.

## Data Availability

The original contributions presented in the study are included in the article/Supplementary Material, further inquiries can be directed to the corresponding author.
