# Supplementary material for: Pharmacovigilance analysis of central nervous system adverse events associated with sevoflurane and drug interactions: a disproportionality study based on the FDA adverse event reporting system (FAERS) database
Source: Front Pharmacol. 2026 Jun 30;17:1789072. doi: 10.3389/fphar.2026.1789072 (PMC13365002; doi:10.3389/fphar.2026.1789072)
Supplement: Supplementary file 1 [file Table1.DOCX]

**Supplementary Table S1. Comparison of IOR and Ω Shrinkage Measure for DDI Signal Detection (PS-Restricted Cohort)**

| **Drug** | **Adverse Event** | **N** | **IOR (95% CI)** | **IOR Signal** | **Ω Shrinkage (95% CI)** | **Ω Signal** | **Final Adjudication** |
| --- | --- | --- | --- | --- | --- | --- | --- |
| Morphine | Dystonia | 8 | 9.46 (3.57–25.08) | Moderate | 4.08 (−0.03–8.19) | No | Filtered by Ω (False Positive) |
| Fentanyl | Dystonia | 12 | 2.96 (1.22–7.17) | Strong | 4.63 (0.57–8.70) | Yes | Confirmed Signal |
| Propofol | Confusional state | 15 | 2.56 (1.02–6.39) | Strong | 4.94 (0.90–8.99) | Yes | Confirmed Signal |
| Morphine | Dyskinesia | 3 | 3.06 (0.81–11.51) | No | 2.80 (−1.46–7.06) | No | No Signal |
| Morphine | Tremor | 1 | 4.32 (0.63–29.63) | No | 1.54 (−3.02–6.10) | No | No Signal |
| Fentanyl | Dyskinesia | 11 | 1.57 (0.59–4.18) | No | 4.51 (0.44–8.57) | Yes | Ω-Unique Signal |
| Fentanyl | Seizure | 5 | 1.61 (0.53–4.91) | No | 3.42 (−0.70–7.54) | No | No Signal |
| Fentanyl | Epilepsy | 2 | 2.36 (0.51–10.83) | No | 2.31 (−2.06–6.67) | No | No Signal |
| Sufentanil | Confusional state | 11 | 2.95 (0.46–18.95) | No | 4.52 (0.44–8.61) | Yes | Ω-Unique Signal |
| Fentanyl | Status epilepticus | 6 | 1.34 (0.41–4.40) | No | 3.70 (−0.45–7.84) | No | No Signal |
| Morphine | Agitation | 4 | 1.06 (0.36–3.14) | No | 3.15 (−1.04–7.34) | No | No Signal |
| Propofol | Lethargy | 3 | 1.42 (0.31–6.38) | No | 2.80 (−1.47–7.07) | No | No Signal |
| Morphine | Status epilepticus | 1 | 1.83 (0.29–11.69) | No | 1.58 (−3.03–6.20) | No | No Signal |
| Remifentanil | Confusional state | 2 | 1.64 (0.25–10.96) | No | 2.32 (−2.06–6.70) | No | No Signal |
| Ketamine | Confusional state | 1 | 1.47 (0.24–9.13) | No | 1.58 (−3.03–6.20) | No | No Signal |
| Propofol | Agitation | 27 | 0.51 (0.26–1.01) | No | 5.78 (1.75–9.80) | Yes | Ω-Unique Signal |
| Propofol | Dystonia | 22 | 0.35 (0.16–0.74) | No | 5.49 (1.45–9.53) | Yes | Ω-Unique Signal |
| Propofol | Convulsion | 14 | 0.15 (0.08–0.29) | No | 4.85 (0.79–8.91) | Yes | Ω-Unique Signal |
| Fentanyl | Delirium | 12 | 0.38 (0.17–0.85) | No | 4.63 (0.57–8.69) | Yes | Ω-Unique Signal |
| Propofol | Somnolence | 12 | 0.55 (0.27–1.15) | No | 4.63 (0.57–8.69) | Yes | Ω-Unique Signal |

*This table evaluates all drug–event pairs with IOR > 1 (top 15 by IOR) as well as additional pairs identified as Ω-unique signals, using the PS-restricted cohort.*

*Abbreviations: IOR, Interaction Reporting Odds Ratio; CI, Confidence Interval; N, number of co-reported cases; Ω, Omega shrinkage measure.*

*IOR Signal criteria: Strong (IOR lower CI > 1, N ≥ 10); Moderate (IOR lower CI > 1, 5 ≤ N < 10); Exploratory (IOR lower CI > 1, 3 ≤ N < 5).*

*Ω Signal criteria: Significant interaction confirmed if Ω lower 95% CI > 0 (Noguchi et al., Pharm Res. 2020;37(5):86).*

*Final Adjudication:*

- *"Confirmed Signal": Detected by both frequency (IOR) and Bayesian shrinkage (Ω) models.*
- *"Filtered by Ω (False Positive)": IOR-positive signal not confirmed by the conservative Ω algorithm, suggesting potential inflation due to small sample size or high baseline reporting.*
- *"Ω-Unique Signal": Underlying interaction signals uncovered by the Ω algorithm that were missed by frequency-based IOR thresholding.*

*Data source: FDA Adverse Event Reporting System (FAERS), 2004Q1–2025Q2.*

**Supplementary Table S2. Multi-Method Disproportionality Analysis: ROR, EBGM, and IC**

| **Drug** | **Adverse Event** | **N** | **ROR (95% CI lower)** | **EBGM (EB05)** | **IC (IC025)** | **Concordance** |
| --- | --- | --- | --- | --- | --- | --- |
| Propofol | Agitation | 27 | 5.38 (3.69) | 4.83 (3.42) | 2.27 (1.73) | Strong (3/3) |
| Propofol | Dystonia | 22 | 16.10 (10.62) | 11.72 (7.98) | 3.55 (2.96) | Strong (3/3) |
| Propofol | Convulsion | 14 | 2.72 (1.62) | 2.47 (1.51) | 1.30 (0.56) | Strong (3/3) |
| Fentanyl | Delirium | 12 | 7.80 (4.46) | 5.89 (3.44) | 2.56 (1.76) | Strong (3/3) |
| Fentanyl | Dystonia | 12 | 13.22 (7.57) | 8.58 (5.01) | 3.10 (2.30) | Strong (3/3) |
| Sufentanil | Confusional state | 11 | 3.84 (2.14) | 3.23 (1.84) | 1.69 (0.86) | Strong (3/3) |
| Fentanyl | Dyskinesia | 11 | 6.23 (3.48) | 4.86 (2.77) | 2.28 (1.45) | Strong (3/3) |
| Propofol | Dyskinesia | 11 | 4.18 (2.34) | 3.52 (2.00) | 1.82 (0.98) | Strong (3/3) |
| Fentanyl | Convulsion | 9 | 2.64 (1.39) | 2.31 (1.23) | 1.21 (0.29) | Strong (3/3) |
| Fentanyl | Agitation | 9 | 2.73 (1.44) | 2.38 (1.26) | 1.25 (0.33) | Strong (3/3) |
| Propofol | Delirium | 8 | 3.55 (1.81) | 2.93 (1.49) | 1.55 (0.58) | Strong (3/3) |
| Propofol | Cognitive disorder | 8 | 2.70 (1.38) | 2.32 (1.19) | 1.22 (0.25) | Strong (3/3) |
| Morphine | Dystonia | 8 | 66.64 (33.25) | 13.38 (6.83) | 3.74 (2.77) | Strong (3/3) |
| Propofol | Coma | 8 | 2.50 (1.27) | 2.17 (1.11) | 1.12 (0.15) | Strong (3/3) |
| Remifentanil | Convulsion | 8 | 4.49 (2.28) | 3.52 (1.79) | 1.81 (0.84) | Strong (3/3) |
| Sufentanil | Coma | 7 | 8.21 (3.98) | 5.25 (2.54) | 2.39 (1.36) | Strong (3/3) |
| Fentanyl | Status epilepticus | 6 | 12.44 (5.75) | 6.34 (2.87) | 2.66 (1.55) | Strong (3/3) |
| Propofol | Status epilepticus | 6 | 8.37 (3.87) | 5.08 (2.30) | 2.34 (1.24) | Strong (3/3) |
| Remifentanil | Status epilepticus | 6 | 23.61 (10.89) | 8.35 (3.78) | 3.06 (1.95) | Strong (3/3) |
| Morphine | Agitation | 4 | 9.32 (3.64) | 4.53 (1.67) | 2.18 (0.84) | Strong (3/3) |
| Morphine | Dyskinesia | 3 | 13.52 (4.67) | 4.58 (1.42) | 2.20 (0.69) | Strong (3/3) |
| Sufentanil | Hypertonia | 2 | 20.63 (5.95) | 4.02 (0.92) | 2.01 (0.22) | Moderate (2/3) |
| Fentanyl | Coma | 5 | 2.40 (1.04) | 1.96 (0.82) | 0.97 (−0.23) | Weak (1/3) |
| Sufentanil | Convulsion | 4 | 3.10 (1.22) | 2.30 (0.85) | 1.20 (−0.13) | Weak (1/3) |
| Sufentanil | Agitation | 4 | 3.21 (1.27) | 2.35 (0.87) | 1.24 (−0.10) | Weak (1/3) |
| Sufentanil | Cognitive disorder | 3 | 4.09 (1.43) | 2.58 (0.80) | 1.37 (−0.15) | Weak (1/3) |
| Ketamine | Agitation | 2 | 4.19 (1.20) | 2.27 (0.52) | 1.18 (−0.60) | Weak (1/3) |
| Sufentanil | Status epilepticus | 1 | 7.08 (1.42) | 2.11 (0.25) | 1.07 (−1.23) | Weak (1/3) |
| Propofol | Stupor | 1 | 6.41 (1.29) | 2.04 (0.24) | 1.03 (−1.28) | Weak (1/3) |
| Remifentanil | Hypertonia | 1 | 9.40 (1.89) | 2.27 (0.27) | 1.18 (−1.12) | Weak (1/3) |
| Fentanyl | Hypertonia | 1 | 4.97 (1.00) | 1.87 (0.22) | 0.90 (−1.40) | Weak (1/3) |
| Morphine | Status epilepticus | 1 | 20.26 (4.05) | 2.61 (0.31) | 1.39 (−0.92) | Weak (1/3) |
| Morphine | Sedation | 1 | 9.22 (1.84) | 2.26 (0.27) | 1.18 (−1.13) | Weak (1/3) |

Multi-method concordance: Strong (3/3) = 21; Moderate (2/3) = 1; Weak (1/3) = 11; None (0/3) = 34.

Only drug-event pairs with ≥ 1 positive method are shown.

Abbreviations: ROR, Reporting Odds Ratio; EBGM, Empirical Bayesian Geometric Mean; EB05, lower 5th percentile of EBGM posterior; IC, Information Component; IC025, lower 2.5th percentile of IC posterior.

Data source: FDA Adverse Event Reporting System (FAERS), 2004Q1–2025Q2.

Signal criteria: ROR lower 95% CI > 1; EB05 > 1 (DuMouchel, 1999); IC025 > 0 (Bate et al., 1998).

EBGM applies Bayesian shrinkage toward the null, inherently penalizing estimates from small counts.

**Supplementary Table S3. Consolidated Summary of Statistically Significant IOR Signals Validated by the Ω Shrinkage Measure (PS-Restricted Cohort)**

| **Drug** | **Adverse Event** | **N** | **Frequency Model: IOR (95% CI)** | **IOR Signal Strength** | **Bayesian DDI Model: Ω Shrinkage (95% CI)** | **Ω Validation Status** | **Final Adjudication** |
| --- | --- | --- | --- | --- | --- | --- | --- |
| Morphine | Dystonia | 8 | 9.46 (3.57–25.08) | Moderate | 4.08 (−0.03–8.19) | Not Validated (Ω lower CI < 0) | Filtered by Ω |
| Fentanyl | Dystonia | 12 | 2.96 (1.22–7.17) | Strong | 4.63 (0.57–8.70) | Validated (Ω lower CI > 0) | True Synergistic Signal |
| Propofol | Confusional state | 15 | 2.56 (1.02–6.39) | Strong | 4.94 (0.90–8.99) | Validated (Ω lower CI > 0) | True Synergistic Signal |

*This table evaluates the three IOR-positive combinations identified in the PS-restricted primary analysis using the Bayesian Ω shrinkage measure for drug-drug interactions.*

*Abbreviations: IOR, Interaction Reporting Odds Ratio; CI, Confidence Interval; Ω, Omega shrinkage measure.*

*Data source: FDA Adverse Event Reporting System (FAERS), 2004Q1–2025Q2.*

*Signal criteria: IOR lower 95% CI > 1; Ω lower 95% CI > 0 (Noguchi et al., Pharm Res. 2020;37(5):86).*

*IOR Signal Strength: Strong (IOR lower CI > 1, N ≥ 10); Moderate (IOR lower CI > 1, 5 ≤ N < 10).*

*Final Adjudication:*

- *"Filtered by Ω": The IOR signal was not confirmed by the conservative Ω algorithm, suggesting potential inflation due to small sample size (N = 8) or confounding from morphine's intrinsic propensity for dystonia reporting. However, this signal achieved Ω validation under the role-independent framework with larger sample size (N = 19; see Supplementary Table S6), indicating that the PS-restricted Ω non-validation likely reflects sample size limitations rather than absence of a true interaction.*
- *"True Synergistic Signal": The interaction signal remained robust and statistically significant under the strict penalty of the Bayesian Ω shrinkage measure, supporting a genuine supra-additive pharmacodynamic interaction.*

**Supplementary Table S4. Distribution of Interaction Signals Across Role Combinations (Descriptive Analysis for key Drug-Event Pairs)**

| **Drug-Event Pair** | **Total Combo Cases** | **Sevo_PS Drug_PS** | **Sevo_PS Drug_NonPS** | **Sevo_NonPS Drug_PS** | **Sevo_NonPS Drug_NonPS** |
| --- | --- | --- | --- | --- | --- |
| Fentanyl–Dystonia | 91 | 0 (0.0%) | 12 (13.2%) | 6 (6.6%) | 73 (80.2%) |
| Morphine–Dystonia | 19 | 0 (0.0%) | 9 (47.4%) | 3 (15.8%) | 7 (36.8%) |
| Propofol–Confusional state | 57 | 0 (0.0%) | 15 (26.3%) | 4 (7.0%) | 38 (66.7%) |
| Propofol–Dystonia | 100 | 0 (0.0%) | 22 (22.0%) | 9 (9.0%) | 69 (69.0%) |

*This descriptive analysis demonstrates that interaction signals are distributed across multiple role combinations, validating that PS-only restriction may miss substantial signals. This table only showed four Drug-Event Pairs as example.*

*Notably, no cases of any key drug–event pair were observed in the PS–PS combination, confirming that simultaneous dual-PS attribution is rare in FAERS for these combinations.*

*Percentages represent the proportion of cases with the event falling into each role combination category.*

*Abbreviations: PS, Primary Suspect; NonPS, includes Secondary Suspect, Concomitant, and Interacting roles.*

*Data source: FDA Adverse Event Reporting System (FAERS), 2004Q1–2025Q2.*

**Supplementary Table S5. Role-Independent DDI Analysis Using All Drug Roles: Comparison of IOR and Ω Shrinkage Measure**

| **Drug–Event Pair** | **N (All Roles)** | **IOR (95% CI)** | **IOR Signal** | **Ω Shrinkage (95% CI)** | **Ω Signal** | **Adjudication** |
| --- | --- | --- | --- | --- | --- | --- |
| Alfentanil–Amnesia | 4 | 10.75 (2.86–40.37) | Yes | 3.17 (-1.04–7.38) | No | Filtered by Ω (False Positive) |
| Morphine–Status epilepticus | 6 | 2.98 (1.24–7.15) | Yes | 3.69 (−0.45–7.82) | No | Filtered by Ω (False Positive) |
| Morphine–Tremor | 15 | 2.73 (1.54–4.86) | Yes | 4.77 (0.95–8.60) | Yes | Confirmed Signal |
| Sufentanil–Confusional state | 18 | 2.58 (1.45–4.59) | Yes | 5.21 (1.16–9.26) | Yes | Confirmed Signal |
| Remifentanil–Tremor | 13 | 2.40 (1.21–4.80) | Yes | — | No | Filtered by Ω |
| Morphine–Dystonia | 19 | 1.80 (1.09–2.97) | Yes | 5.26 (1.24–9.28) | Yes | Confirmed Signal |
| Propofol–Tremor | 36 | 1.76 (1.13–2.74) | Yes | 6.14 (2.18–10.10) | Yes | Confirmed Signal |
| Propofol–Lethargy | 9 | 1.39 (0.61–3.13) | No | 4.23 (0.15–8.31) | Yes | Ω-Unique Signal |
| Fentanyl–Tremor | 36 | 1.19 (0.77–1.82) | No | 5.93 (2.25–9.61) | Yes | Ω-Unique Signal |
| Propofol–Somnolence | 74 | 1.16 (0.85–1.58) | No | 7.16 (3.23–11.09) | Yes | Ω-Unique Signal |
| Propofol–Confusional state | 57 | 1.06 (0.75–1.51) | No | 6.80 (2.85–10.75) | Yes | Ω-Unique Signal |
| Fentanyl–Dystonia | 91 | 1.04 (0.77–1.40) | No | 7.48 (3.52–11.44) | Yes | Ω-Unique Signal |
| Propofol–Cognitive disorder | 11 | 1.01 (0.46–2.21) | No | 4.51 (0.44–8.58) | Yes | Ω-Unique Signal |
| Morphine–Agitation | 21 | 0.96 (0.60–1.53) | No | 5.34 (1.41–9.27) | Yes | Ω-Unique Signal |
| Remifentanil–Confusional state | 11 | 0.81 (0.41–1.60) | No | 4.52 (0.44–8.60) | Yes | Ω-Unique Signal |
| Fentanyl–Dyskinesia | 54 | 0.79 (0.55–1.12) | No | 6.70 (2.78–10.62) | Yes | Ω-Unique Signal |
| Fentanyl–Seizure | 15 | 0.70 (0.40–1.25) | No | 4.79 (0.94–8.63) | Yes | Ω-Unique Signal |
| Propofol–Epilepsy | 17 | 0.54 (0.29–1.00) | No | 5.12 (1.08–9.16) | Yes | Ω-Unique Signal |
| Fentanyl–Somnolence | 62 | 0.53 (0.39–0.72) | No | 6.66 (3.05–10.27) | Yes | Ω-Unique Signal |
| Fentanyl–Encephalopathy | 23 | 0.52 (0.31–0.86) | No | 5.51 (1.53–9.49) | Yes | Ω-Unique Signal |
| Morphine–Confusional state | 10 | 0.51 (0.27–0.98) | No | 4.22 (0.35–8.08) | Yes | Ω-Unique Signal |
| Fentanyl–Sedation | 15 | 0.50 (0.26–0.98) | No | 4.91 (0.91–8.92) | Yes | Ω-Unique Signal |
| Fentanyl–Status epilepticus | 14 | 0.48 (0.25–0.90) | No | 4.84 (0.80–8.88) | Yes | Ω-Unique Signal |
| Fentanyl–Confusional state | 33 | 0.43 (0.29–0.65) | No | 5.81 (2.12–9.49) | Yes | Ω-Unique Signal |
| Propofol–Seizure | 27 | 0.43 (0.27–0.69) | No | 5.75 (1.76–9.75) | Yes | Ω-Unique Signal |
| Fentanyl–Agitation | 49 | 0.39 (0.28–0.54) | No | 6.50 (2.65–10.35) | Yes | Ω-Unique Signal |
| Propofol–Dyskinesia | 52 | 0.37 (0.26–0.54) | No | 6.70 (2.70–10.70) | Yes | Ω-Unique Signal |
| Fentanyl–Convulsion | 23 | 0.37 (0.24–0.59) | No | 5.42 (1.56–9.29) | Yes | Ω-Unique Signal |
| Fentanyl–Coma | 41 | 0.34 (0.24–0.49) | No | 6.29 (2.38–10.20) | Yes | Ω-Unique Signal |
| Propofol–Convulsion | 33 | 0.34 (0.23–0.51) | No | 6.04 (2.05–10.04) | Yes | Ω-Unique Signal |
| Propofol–Agitation | 84 | 0.31 (0.23–0.41) | No | 7.38 (3.40–11.36) | Yes | Ω-Unique Signal |
| Fentanyl–Delirium | 32 | 0.30 (0.20–0.47) | No | 5.96 (2.01–9.91) | Yes | Ω-Unique Signal |
| Propofol–Coma | 51 | 0.29 (0.20–0.41) | No | 6.67 (2.67–10.67) | Yes | Ω-Unique Signal |
| Sufentanil–Agitation | 9 | 0.29 (0.14–0.59) | No | 4.25 (0.15–8.35) | Yes | Ω-Unique Signal |
| Sufentanil–Coma | 10 | 0.22 (0.11–0.43) | No | 4.39 (0.30–8.48) | Yes | Ω-Unique Signal |
| Propofol–Delirium | 31 | 0.22 (0.14–0.35) | No | 5.97 (1.95–9.98) | Yes | Ω-Unique Signal |
| Propofol–Dystonia | 100 | 0.21 (0.15–0.27) | No | 7.64 (3.64–11.64) | Yes | Ω-Unique Signal |
| Propofol–Encephalopathy | 25 | 0.19 (0.11–0.31) | No | 5.66 (1.64–9.69) | Yes | Ω-Unique Signal |
| Remifentanil–Status epilepticus | 11 | 0.18 (0.09–0.39) | No | 4.52 (0.44–8.61) | Yes | Ω-Unique Signal |
| Ketamine–Agitation | 11 | 0.17 (0.09–0.32) | No | 4.52 (0.44–8.60) | Yes | Ω-Unique Signal |
| Propofol–Status epilepticus | 19 | 0.05 (0.03–0.09) | No | 5.28 (1.24–9.33) | Yes | Ω-Unique Signal |

*This table presents the role-independent DDI analysis incorporating all drug roles (Primary Suspect, Secondary Suspect, Concomitant, and Interacting) from the full FAERS database, with dual-method evaluation using both IOR and Ω shrinkage measure.*

*IOR (Interaction Reporting Odds Ratio) = ROR_AB / (ROR_A × ROR_B), where all RORs were calculated using cases with any role designation.*

*Abbreviations: IOR, Interaction Reporting Odds Ratio; CI, Confidence Interval; Ω, Omega shrinkage measure.*

*Data source: FDA Adverse Event Reporting System (FAERS), 2004Q1–2025Q2.*

*Signal criteria: IOR lower 95% CI > 1; Ω lower 95% CI > 0.*

*Final Adjudication:*

- *"Confirmed Signal": Detected robustly by both the frequency model (IOR) and the Bayesian shrinkage model (Ω).*
- *"Filtered by Ω (False Positive)": IOR-positive signal not confirmed by the conservative Ω algorithm.*
- *"Ω-Unique Signal": Interaction signals uncovered by the Ω algorithm that were missed by the frequency-based IOR due to signal dilution from high baseline disproportionality across all roles. This pattern is expected: when a drug exhibits high disproportionality for a given event across all role designations, the ROR_B denominator inflates, suppressing IOR below significance. The Ω shrinkage measure, which employs a different computational framework, is not subject to this ratio-of-ratios dilution effect.*

*Of the seven IOR-positive signals, four were confirmed by Ω (Morphine–Tremor, Sufentanil–Confusional state, Morphine–Dystonia, Propofol–Tremor), while three were filtered (Alfentanil–Amnesia*, *Morphine–Status epilepticus, Remifentanil–Tremor). An additional 28 Ω-unique signals were identified, demonstrating the complementary value of the Bayesian DDI-specific method.*

**Supplementary Table S6. Consolidated Summary of Key DDI Signals Validated by Ω Shrinkage Measure in Full Database (All Roles)**

| **Drug** | **Adverse Event** | **N (All Roles)** | **Frequency Model: All-Roles IOR (95% CI)** | **IOR Signal** | **Bayesian DDI Model: All-Roles Ω (95% CI)** | **Ω Validation Status** | **Final Adjudication** |
| --- | --- | --- | --- | --- | --- | --- | --- |
| Morphine | Dystonia | 19 | 1.80 (1.09–2.97) | Yes | 5.26 (1.24–9.28) | Validated (Ω lower CI > 0) | True Synergistic Signal |
| Fentanyl | Dystonia | 91 | 1.04 (0.77–1.40) | No | 7.48 (3.52–11.44) | Validated (Ω lower CI > 0) | Ω-Unique Signal (IOR diluted) |
| Propofol | Confusional state | 57 | 1.06 (0.75–1.51) | No | 6.80 (2.85–10.75) | Validated (Ω lower CI > 0) | Ω-Unique Signal (IOR diluted) |

*This table validates the three core DDI combinations in the full FAERS database (N = 20,609,096) without restricting drug roles, providing cross-framework comparison with the PS-restricted results (Supplementary Table S3).*

*Abbreviations: IOR, Interaction Reporting Odds Ratio; CI, Confidence Interval; Ω, Omega shrinkage measure.*

*Data source: FDA Adverse Event Reporting System (FAERS), 2004Q1–2025Q2.*

*Signal criteria: IOR lower 95% CI > 1; Ω lower 95% CI > 0.*

*Cross-framework comparison: All three core DDI combinations achieved Ω validation under the role-independent framework. Morphine–Dystonia was confirmed by both IOR and Ω, achieving cross-framework robustness despite its non-validation by Ω in the PS-restricted analysis (N = 8 vs. N = 19). Fentanyl–Dystonia and Propofol–Confusional state demonstrated IOR dilution under all-roles analysis (due to high baseline disproportionality across roles) but were confirmed by the Ω algorithm, demonstrating the complementary value of Bayesian DDI-specific methods for detecting true interaction signals masked by ratio-of-ratios dilution effects.*

**Supplementary Table S7. Vulnerable Population Subgroup Analysis within Sevoflurane Users**

| **Subgroup** | **Adverse Event** | **N (Vuln)** | **ROR (95% CI) Vuln vs. Non-Vuln** |
| --- | --- | --- | --- |
| Parkinsonism | Dystonia | 19 | 12.20 (7.33–20.30) |
| Parkinsonism | Dyskinesia | 11 | 10.38 (5.46–19.74) |
| Parkinsonism | Convulsion | 4 | 2.75 (1.06–7.17) |
| Parkinsonism | Coma | 9 | 6.46 (3.25–12.82) |
| Parkinsonism | Somnolence | 5 | 2.94 (1.23–7.02) |
| Parkinsonism | Agitation | 17 | 10.70 (6.29–18.18) |
| Parkinsonism | Confusional state | 10 | 8.91 (4.58–17.33) |
| Parkinsonism | Seizure | 4 | 5.51 (2.07–14.63) |
| Parkinsonism | Status epilepticus | 1 | 3.09 (0.59–16.06) |
| Neurocognitive | Coma | 1 | 2.58 (0.50–13.19) |
| Neurocognitive | Agitation | 1 | 2.08 (0.41–10.62) |
| Neurocognitive | Delirium | 3 | 11.25 (3.70–34.20) |
| Neurocognitive | Confusional state | 11 | 32.79 (16.54–65.02) |
| Psychiatric | Dystonia | 8 | 1.05 (0.52–2.12) |
| Psychiatric | Dyskinesia | 6 | 1.26 (0.56–2.81) |
| Psychiatric | Convulsion | 1 | 0.21 (0.04–1.07) |
| Psychiatric | Coma | 13 | 2.25 (1.26–4.02) |
| Psychiatric | Somnolence | 16 | 2.37 (1.40–4.00) |
| Psychiatric | Agitation | 25 | 3.82 (2.46–5.95) |
| Psychiatric | Delirium | 9 | 2.80 (1.40–5.64) |
| Psychiatric | Confusional state | 19 | 4.44 (2.66–7.40) |
| Psychiatric | Seizure | 1 | 0.41 (0.08–2.09) |

*This post-hoc subgroup analysis identifies vulnerable populations based on keyword matching within the INDI (Indication for use) and REAC (Reaction) tables of the FAERS database.*

*The "Non-Vuln" group serves as the reference cohort, consisting of sevoflurane users without the specific baseline condition identified in the respective subgroup.*

*Abbreviations: ROR, Reporting Odds Ratio; CI, Confidence Interval; Vuln, Vulnerable Population.*

*Data source: FDA Adverse Event Reporting System (FAERS), 2004Q1–2025Q2.*

*Statistical Interpretation: An ROR > 1 with a lower 95% CI > 1 indicates a significantly higher reporting frequency of the specific CNS event within that vulnerable subgroup compared to the non-vulnerable sevoflurane user cohort. This supports a "double-hit" model where baseline neurological vulnerability may lower the threshold for drug-induced CNS adverse events.*

**Supplementary Table S8. Time-to-Event (TTE) Distribution for Sevoflurane-Related CNS Events**

| **Time Interval** | **Number of Cases (N)** | **Percentage (%)** |
| --- | --- | --- |
| 0 days (Same day) | 2,238 | 70.5% |
| 1–7 days | 601 | 18.9% |
| 8–30 days | 136 | 4.3% |
| > 30 days | 201 | 6.3% |

*Total reports with valid start and event dates: N = 3,176.*

*Time-to-Event (TTE) is calculated as the difference between the adverse event date (EVENT_DT) and the drug start date (START_DT).*

*Data source: FDA Adverse Event Reporting System (FAERS), 2004Q1–2025Q2.*

*Interpretation: The high clustering at Day 0 (Same day) supports an acute pharmacodynamic interaction occurring during or immediately after anesthesia.*

**Supplementary Table S9. Sensitivity Analysis: PS-Only vs. PS+SS Expanded Cohort**

| **Drug** | **Adverse Event** | **N (PS)** | **ROR (95% CI) PS-Only** | **N (PS+SS)** | **ROR (95% CI) PS+SS** | **Concordance** |
| --- | --- | --- | --- | --- | --- | --- |
| Fentanyl | Agitation | 9 | 2.73 (1.44–5.17) | 18 | 2.31 (1.46–3.66) | Concordant |
| Fentanyl | Cognitive disorder | 3 | 1.65 (0.58–4.70) | 3 | 0.72 (0.25–2.04) | Concordant |
| Fentanyl | Coma | 5 | 2.40 (1.04–5.54) | 12 | 2.38 (1.36–4.14) | Concordant |
| Fentanyl | Confusional state | 2 | 0.33 (0.09–1.13) | 15 | 0.89 (0.54–1.47) | Concordant |
| Fentanyl | Convulsion | 9 | 2.64 (1.39–5.00) | 13 | 1.63 (0.95–2.78) | Discordant |
| Fentanyl | Delirium | 12 | 7.80 (4.46–13.62) | 21 | 5.83 (3.81–8.91) | Concordant |
| Fentanyl | Dyskinesia | 11 | 6.23 (3.48–11.14) | 34 | 8.18 (5.84–11.45) | Concordant |
| Fentanyl | Dystonia | 12 | 13.22 (7.57–23.11) | 38 | 17.86 (12.98–24.57) | Concordant |
| Fentanyl | Encephalopathy | 2 | 2.15 (0.62–7.44) | 9 | 3.57 (1.89–6.75) | Discordant |
| Fentanyl | Epilepsy | 2 | 1.76 (0.51–6.08) | 3 | 1.07 (0.38–3.06) | Concordant |
| Fentanyl | Hallucination | 2 | 0.74 (0.21–2.54) | 4 | 0.58 (0.23–1.45) | Concordant |
| Fentanyl | Hypertonia | 1 | 4.97 (1.00–24.69) | 2 | 3.61 (1.04–12.49) | Concordant |
| Fentanyl | Lethargy | 1 | 0.56 (0.11–2.77) | 1 | 0.24 (0.05–1.21) | Concordant |
| Fentanyl | Sedation | 2 | 2.17 (0.63–7.50) | 7 | 2.84 (1.38–5.81) | Discordant |
| Fentanyl | Seizure | 5 | 1.18 (0.51–2.73) | 11 | 1.08 (0.60–1.92) | Concordant |
| Fentanyl | Somnolence | 8 | 0.93 (0.47–1.83) | 39 | 1.91 (1.39–2.61) | Discordant |
| Fentanyl | Status epilepticus | 6 | 12.44 (5.75–26.91) | 11 | 9.58 (5.37–17.11) | Concordant |
| Fentanyl | Tremor | 2 | 0.32 (0.09–1.11) | 5 | 0.31 (0.13–0.71) | Concordant |
| Ketamine | Agitation | 2 | 4.19 (1.20–14.61) | 9 | 5.13 (2.70–9.76) | Concordant |
| Ketamine | Coma | 1 | 3.81 (0.76–19.00) | 1 | 1.21 (0.24–6.03) | Concordant |
| Ketamine | Confusional state | 1 | 1.15 (0.23–5.74) | 5 | 1.36 (0.59–3.14) | Concordant |
| Ketamine | Somnolence | 1 | 0.96 (0.19–4.77) | 3 | 0.71 (0.25–2.04) | Concordant |
| Morphine | Agitation | 4 | 9.32 (3.64–23.85) | 9 | 7.20 (3.78–13.71) | Concordant |
| Morphine | Confusional state | 1 | 1.40 (0.28–6.98) | 3 | 1.20 (0.42–3.44) | Concordant |
| Morphine | Convulsion | 1 | 2.93 (0.59–14.66) | 3 | 2.52 (0.88–7.22) | Concordant |
| Morphine | Dyskinesia | 3 | 13.52 (4.67–39.09) | 3 | 4.90 (1.71–14.04) | Concordant |
| Morphine | Dystonia | 8 | 66.64 (33.25–133.54) | 11 | 32.15 (17.86–57.86) | Concordant |
| Morphine | Sedation | 1 | 9.22 (1.84–46.11) | 1 | 3.38 (0.68–16.78) | Discordant |
| Morphine | Status epilepticus | 1 | 20.26 (4.05–101.33) | 5 | 27.52 (11.85–63.90) | Concordant |
| Morphine | Tremor | 1 | 1.37 (0.27–6.85) | 1 | 0.50 (0.10–2.50) | Concordant |
| Propofol | Agitation | 27 | 5.38 (3.69–7.85) | 47 | 4.27 (3.21–5.69) | Concordant |
| Propofol | Amnesia | 3 | 0.74 (0.26–2.12) | 3 | 0.34 (0.12–0.98) | Concordant |
| Propofol | Cognitive disorder | 8 | 2.70 (1.38–5.30) | 11 | 1.68 (0.94–3.00) | Discordant |
| Propofol | Coma | 8 | 2.50 (1.27–4.91) | 15 | 2.10 (1.27–3.46) | Concordant |
| Propofol | Confusional state | 15 | 1.38 (0.84–2.28) | 43 | 1.80 (1.33–2.42) | Discordant |
| Propofol | Convulsion | 14 | 2.72 (1.62–4.56) | 25 | 2.20 (1.49–3.25) | Concordant |
| Propofol | Delirium | 8 | 3.55 (1.81–6.96) | 17 | 3.37 (2.11–5.39) | Concordant |
| Propofol | Dyskinesia | 11 | 4.18 (2.34–7.48) | 32 | 5.47 (3.87–7.73) | Concordant |
| Propofol | Dystonia | 22 | 16.10 (10.62–24.43) | 44 | 14.69 (10.92–19.75) | Concordant |
| Propofol | Encephalopathy | 1 | 0.87 (0.18–4.31) | 9 | 2.54 (1.34–4.81) | Discordant |
| Propofol | Epilepsy | 4 | 2.14 (0.85–5.39) | 16 | 3.62 (2.23–5.87) | Discordant |
| Propofol | Hallucination | 3 | 0.69 (0.24–1.98) | 4 | 0.41 (0.16–1.04) | Concordant |
| Propofol | Lethargy | 3 | 0.88 (0.31–2.51) | 4 | 0.52 (0.21–1.31) | Concordant |
| Propofol | Seizure | 7 | 1.09 (0.53–2.23) | 20 | 1.37 (0.89–2.12) | Concordant |
| Propofol | Somnolence | 12 | 0.93 (0.53–1.61) | 48 | 1.67 (1.26–2.21) | Discordant |
| Propofol | Status epilepticus | 6 | 8.37 (3.87–18.09) | 15 | 9.22 (5.59–15.19) | Concordant |
| Propofol | Stupor | 1 | 6.41 (1.29–31.77) | 1 | 2.95 (0.60–14.64) | Discordant |
| Propofol | Tremor | 3 | 0.30 (0.11–0.87) | 6 | 0.26 (0.12–0.56) | Concordant |
| Remifentanil | Cognitive disorder | 1 | 1.33 (0.27–6.60) | 1 | 0.67 (0.13–3.31) | Concordant |
| Remifentanil | Confusional state | 2 | 0.62 (0.18–2.15) | 2 | 0.31 (0.09–1.08) | Concordant |
| Remifentanil | Convulsion | 8 | 4.49 (2.28–8.84) | 5 | 1.44 (0.62–3.34) | Discordant |
| Remifentanil | Delirium | 1 | 1.75 (0.35–8.67) | 2 | 1.46 (0.42–5.06) | Concordant |
| Remifentanil | Dyskinesia | 2 | 2.53 (0.73–8.78) | 4 | 2.29 (0.91–5.79) | Concordant |
| Remifentanil | Epilepsy | 2 | 3.32 (0.96–11.52) | 3 | 2.34 (0.82–6.68) | Concordant |
| Remifentanil | Hypertonia | 1 | 9.40 (1.89–46.68) | — | — | Discordant |
| Remifentanil | Seizure | 2 | 1.01 (0.29–3.51) | 3 | 0.71 (0.25–2.03) | Concordant |
| Remifentanil | Status epilepticus | 6 | 23.61 (10.89–51.20) | 10 | 19.15 (10.42–35.18) | Concordant |
| Sufentanil | Agitation | 4 | 3.21 (1.27–8.12) | 5 | 2.18 (0.94–5.06) | Discordant |
| Sufentanil | Cognitive disorder | 3 | 4.09 (1.43–11.73) | 6 | 4.26 (1.97–9.22) | Concordant |
| Sufentanil | Coma | 7 | 8.21 (3.98–16.91) | 3 | 2.11 (0.74–6.04) | Discordant |
| Sufentanil | Confusional state | 11 | 3.84 (2.14–6.91) | 17 | 3.26 (2.03–5.24) | Concordant |
| Sufentanil | Convulsion | 4 | 3.10 (1.22–7.85) | 5 | 2.11 (0.91–4.89) | Discordant |
| Sufentanil | Delirium | 1 | 2.29 (0.46–11.39) | 3 | 3.00 (1.05–8.57) | Discordant |
| Sufentanil | Dystonia | 1 | 3.89 (0.78–19.32) | 1 | 2.17 (0.44–10.78) | Concordant |
| Sufentanil | Hypertonia | 2 | 20.63 (5.95–71.56) | 2 | 11.51 (3.32–39.86) | Concordant |
| Sufentanil | Somnolence | 2 | 0.68 (0.20–2.35) | — | — | Discordant |
| Sufentanil | Status epilepticus | 1 | 7.08 (1.42–35.19) | 1 | 3.96 (0.80–19.64) | Discordant |

Signal concordance: 48/67 (71.6%) drug-event pairs showed concordant signal status between PS-only and PS+SS analyses.

Abbreviations: PS, Primary Suspect; SS, Secondary Suspect; ROR, Reporting Odds Ratio; CI, Confidence Interval.

Data source: FDA Adverse Event Reporting System (FAERS), 2004Q1–2025Q2.

PS-only cohort: N = 4,123; PS+SS cohort: N = 6,771 (64.2% increase).

Signal criterion: ROR lower 95% CI > 1.

**Supplementary Table S10. Leave-One-Out Sensitivity Analysis Comparing IOR and Ω Shrinkage Measure**

| **Drug–Event Pair** | **Cases Removed** | **Remaining N** | **IOR (95% CI)** | **IOR Signal** | **Ω Shrinkage (95% CI)** | **Ω Signal** | **Adjudication** |
| --- | --- | --- | --- | --- | --- | --- | --- |
| **Morphine–Dystonia** | 1 | 7 | 8.28 (3.03–22.63) | Yes | 3.90 (−0.22–8.02) | No | Lost by Ω, retained by IOR |
|  | 2 | 6 | 7.12 (2.51–20.20) | Yes | 3.69 (−0.45–7.84) | No | Lost by Ω, retained by IOR |
|  | 3 | 5 | 5.97 (2.00–17.82) | Yes | 3.45 (−0.72–7.62) | No | Lost by Ω, retained by IOR |
|  | 4 | 4 | 4.85 (1.52–15.48) | Yes | 3.16 (−1.04–7.37) | No | Lost by Ω, retained by IOR |
|  | 5 | 3 | 3.74 (1.06–13.20) | Yes | 2.80 (−1.47–7.07) | No | Lost by Ω, retained by IOR |
|  | 6 | 2 | 2.65 (0.64–10.97) | No | 2.32 (−2.06–6.69) | No | Signal Lost |
|  | 7 | 1 | 1.58 (0.27–9.25) | No | 1.58 (−3.03–6.19) | No | Signal Lost |
| **Fentanyl–Dystonia** | 1 | 11 | 2.72 (1.10–6.73) | Yes | 4.51 (0.44–8.59) | Yes | Confirmed |
|  | 2 | 10 | 2.48 (0.99–6.21) | No | 4.38 (0.30–8.46) | Yes | Lost by IOR, retained by Ω |
|  | 3 | 9 | 2.24 (0.87–5.77) | No | 4.24 (0.15–8.33) | Yes | Lost by IOR, retained by Ω |
|  | 4 | 8 | 2.00 (0.76–5.26) | No | 4.08 (−0.02–8.18) | No | Signal Lost |
|  | 5 | 7 | 1.76 (0.65–4.77) | No | 3.90 (−0.22–8.02) | No | Signal Lost |
|  | 6 | 6 | 1.53 (0.54–4.33) | No | 3.69 (−0.45–7.83) | No | Signal Lost |
|  | 7 | 5 | 1.29 (0.44–3.78) | No | 3.45 (−0.71–7.62) | No | Signal Lost |
|  | 8 | 4 | 1.05 (0.33–3.34) | No | 3.16 (−1.04–7.36) | No | Signal Lost |
|  | 9 | 3 | 0.82 (0.23–2.92) | No | 2.80 (−1.47–7.06) | No | Signal Lost |
|  | 10 | 2 | 0.58 (0.14–2.40) | No | 2.31 (−2.06–6.68) | No | Signal Lost |
|  | 11 | 1 | 0.35 (0.06–2.04) | No | 1.58 (−3.03–6.18) | No | Signal Lost |
| **Propofol–Confusional state** | 1 | 14 | 2.39 (0.95–6.01) | No | 4.85 (0.80–8.90) | Yes | Lost by IOR, retained by Ω |
|  | 2 | 13 | 2.22 (0.87–5.66) | No | 4.74 (0.69–8.80) | Yes | Lost by IOR, retained by Ω |
|  | 3 | 12 | 2.06 (0.80–5.30) | No | 4.63 (0.57–8.69) | Yes | Lost by IOR, retained by Ω |
|  | 4 | 11 | 1.89 (0.72–4.96) | No | 4.51 (0.44–8.58) | Yes | Lost by IOR, retained by Ω |
|  | 5 | 10 | 1.72 (0.65–4.55) | No | 4.38 (0.30–8.46) | Yes | Lost by IOR, retained by Ω |
|  | 6 | 9 | 1.56 (0.57–4.27) | No | 4.24 (0.15–8.32) | Yes | Lost by IOR, retained by Ω |
|  | 7 | 8 | 1.39 (0.50–3.86) | No | 4.08 (−0.02–8.17) | No | Signal Lost |
|  | 8 | 7 | 1.23 (0.43–3.52) | No | 3.89 (−0.22–8.01) | No | Signal Lost |
|  | 9 | 6 | 1.06 (0.36–3.12) | No | 3.69 (−0.45–7.82) | No | Signal Lost |
|  | 10 | 5 | 0.90 (0.29–2.79) | No | 3.45 (−0.71–7.61) | No | Signal Lost |
|  | 11 | 4 | 0.74 (0.22–2.49) | No | 3.16 (−1.04–7.36) | No | Signal Lost |
|  | 12 | 3 | 0.57 (0.16–2.03) | No | 2.80 (−1.46–7.05) | No | Signal Lost |
|  | 13 | 2 | 0.41 (0.09–1.87) | No | 2.31 (−2.06–6.67) | No | Signal Lost |
|  | 14 | 1 | 0.24 (0.04–1.44) | No | 1.57 (−3.03–6.18) | No | Signal Lost |

*Leave-one-out analysis: cases are sequentially removed from the combination cell (a) of the 2×2 table to assess signal stability under both the frequency-based IOR and Bayesian Ω shrinkage measure.*

*Original IOR values: Morphine–Dystonia = 9.46 (3.57–25.08), N = 8; Fentanyl–Dystonia = 2.96 (1.22–7.17), N = 12; Propofol–Confusional state = 2.56 (1.02–6.39), N = 15.*

*Original Ω values: Morphine–Dystonia = 4.08 (−0.03–8.19); Fentanyl–Dystonia = 4.63 (0.57–8.70); Propofol–Confusional state = 4.94 (0.90–8.99).*

*Abbreviations: IOR, Interaction Reporting Odds Ratio; CI, Confidence Interval; Ω, Omega shrinkage measure.*

*Data source: FDA Adverse Event Reporting System (FAERS), 2004Q1–2025Q2.*

*Signal criteria: IOR lower 95% CI > 1; Ω lower 95% CI > 0.*

*Key findings: For Morphine–Dystonia, IOR retained significance after removing up to 5 cases while Ω was non-significant throughout, consistent with the PS-restricted Ω non-validation reflecting sample size sensitivity. For Fentanyl–Dystonia, Ω demonstrated superior stability over IOR (signal retained at N = 9 vs. N = 11). For Propofol–Confusional state, Ω markedly outperformed IOR (signal retained at N = 9 vs. N = 15, i.e., stable after removing 6 cases vs. loss upon removing 1 case), confirming the Ω algorithm's superior robustness for this borderline-IOR signal.*

**Supplementary Table S11. Main Effect Analysis against Active Comparator Background**

| **Adverse Event** | **Sevo+AE** | **Comp+AE** | **ROR (95% CI)** | **Signal** |
| --- | --- | --- | --- | --- |
| Dystonia | 12 | 41 | 3.14 (1.66–5.92) | Yes |
| Convulsion | 33 | 154 | 2.28 (1.56–3.32) | Yes |
| Somnolence | 24 | 109 | 2.34 (1.50–3.64) | Yes |
| Coma | 25 | 136 | 1.95 (1.27–2.99) | Yes |
| Delirium | 11 | 53 | 2.23 (1.18–4.24) | Yes |

*This sensitivity analysis replaces the full FAERS database background with an active comparator cohort to mitigate confounding by indication and perioperative context.*

*The active comparator background (Comp) consists exclusively of patients reporting desflurane, isoflurane, or propofol as monotherapy (N = 12,689), who share similar surgical indications and healthcare settings with sevoflurane users.*

*Abbreviations: ROR, Reporting Odds Ratio; CI, Confidence Interval; Sevo+AE, sevoflurane cases reporting the adverse event; Comp+AE, comparator cases reporting the adverse event.*

*Data source: FDA Adverse Event Reporting System (FAERS), 2004Q1–2025Q2.*

*Signal criterion: ROR lower 95% CI > 1.*

*Interpretation: Five CNS adverse events retained significant disproportionality signals when evaluated against the active comparator background, supporting the specificity of these signals to sevoflurane beyond the general perioperative context.*

**Supplementary Table S12. Drug-Drug Interaction Analysis against Active Comparator Background Comparing IOR and Ω Shrinkage Measure**

| **Target Drug** | **Adverse Event** | **N (Co-reports)** | **IOR (95% CI)** | **IOR Signal** | **Ω Shrinkage (95% CI)** | **Ω Signal** | **Adjudication** |
| --- | --- | --- | --- | --- | --- | --- | --- |
| Propofol | Agitation | 27 | 1.93 (0.78–4.77) | No | 4.44 (1.87–7.01) | Yes | Ω-Unique Signal |
| Propofol | Dystonia | 22 | 0.13 (0.05–0.34) | No | 3.95 (1.53–6.37) | Yes | Ω-Unique Signal |
| Propofol | Confusional state | 15 | 1.86 (0.57–6.10) | No | 4.00 (1.04–6.96) | Yes | Ω-Unique Signal |
| Propofol | Convulsion | 14 | 10.54 (2.36–47.08) | Yes | 4.33 (0.92–7.73) | Yes | Confirmed Signal |
| Propofol | Somnolence | 12 | 0.18 (0.06–0.51) | No | 3.62 (0.70–6.53) | Yes | Ω-Unique Signal |
| Propofol | Dyskinesia | 11 | 4.57 (1.05–19.91) | Yes | 3.94 (0.57–7.32) | Yes | Confirmed Signal |
| Morphine | Dystonia | 9 | 40.49 (11.34–144.56) | Yes | 2.90 (0.23–5.56) | Yes | Confirmed Signal |
| Propofol | Delirium | 8 | 1.39 (0.30–6.43) | No | 3.63 (0.08–7.17) | Yes | Ω-Unique Signal |
| Propofol | Coma | 8 | 4.00 (0.77–20.62) | No | 3.70 (0.07–7.33) | Yes | Ω-Unique Signal |
| Propofol | Cognitive disorder | 8 | — | No | 3.76 (0.06–7.45) | Yes | Ω-Unique Signal |

*This analysis is strictly limited to the active comparator background pool, consisting only of patients exposed to desflurane, isoflurane, or propofol as monotherapy (N = 12,689).*

*Abbreviations: IOR, Interaction Reporting Odds Ratio; CI, Confidence Interval; Ω, Omega shrinkage measure.*

*Data source: FDA Adverse Event Reporting System (FAERS), 2004Q1–2025Q2.*

*Signal criteria: IOR lower 95% CI > 1; Ω lower 95% CI > 0.*

*Final Adjudication:*

- *"Confirmed Signal": Detected robustly by both frequency (IOR) and Bayesian shrinkage (Ω) models within the active comparator baseline.*
- *"Ω-Unique Signal (Missed by IOR)": The Ω algorithm uncovered interaction signals that the frequency-based IOR model failed to detect due to excessive denominator penalty in the restricted comparator cohort.*

*Key finding: Morphine–Dystonia was confirmed by both IOR (40.49, 95% CI: 11.34–144.56) and Ω (2.90, 95% CI: 0.23–5.56) within the active comparator framework. This provides additional evidence supporting this interaction signal despite its non-validation by Ω in the PS-restricted full-database analysis (Supplementary Table S3), indicating that the signal is robust when evaluated against a clinically appropriate comparator population sharing similar perioperative context.*
